# Linking Microbial Population Succession and DOM Molecular Changes in *Synechococcus*-Derived Organic Matter Addition Incubation

**DOI:** 10.1128/spectrum.02308-21

**Published:** 2022-04-05

**Authors:** Yu Wang, Rui Xie, Yuan Shen, Ruanhong Cai, Chen He, Qi Chen, Weidong Guo, Quan Shi, Nianzhi Jiao, Qiang Zheng

**Affiliations:** a State Key Laboratory for Marine Environmental Science, Institute of Marine Microbes and Ecospheres, College of Ocean and Earth Sciences, College of the Environment and Ecology, Xiamen University, Xiamen, China; b Fujian Key Laboratory of Marine Carbon Sequestration, Xiamen University, Xiamen, China; c State Key Laboratory of Heavy Oil Processing, China University of Petroleum, Beijing, China; d Key Laboratory of Coastal and Wetland Ecosystems, Ministry of Education, Xiamen University, Xiamen, China; University of Texas at San Antonio

**Keywords:** FT-ICR MS, fluorescent DOM, labile DOM, recalcitrant DOM, *Synechococcus*, heterotrophic bacteria

## Abstract

The molecular-level interactions between phytoplankton-derived dissolved organic matter (DOM) and heterotrophic prokaryotes represent a fundamental and yet poorly understood component of the marine elemental cycle. Here, we investigated the degradation of *Synechococcus*-derived organic matter (SynOM) by coastal microorganisms using spectroscopic and ultrahigh-resolution mass spectrometry analyses coupled with high-throughput sequencing. The added SynOM showed a spectrum of reactivity during a 180-day dark incubation experiment. Along with the decrease in DOM bioavailability, the chemical properties of DOM molecules overall showed increases in oxidation state and aromaticity. Both the microbial community and DOM molecular compositions became more homogeneous toward the end of the incubation. The experiment was partitioned into three phases (I, II, and III) based on the total organic carbon consumption rates from 7.0 ± 1.0 to 1.0 ± 0.1 and to 0.1 ± 0.0 μmol C L^−1 ^day^−1^, respectively. Diverse generalists with low abundance were present in all three phases of the experiment, while a few abundant specialists dominated specific phases, suggesting their diverse roles in the transformation of DOM molecules from labile and semilabile to recalcitrant. The changes of organic molecules belonging to CHO, CHNO, and CHOS containing formulas were closely associated with specific microbial populations, suggesting close interactions between the different bacterial metabolic potential for substrates and DOM molecular compositional characteristics. This study sheds light on the interactions between microbial population succession and DOM molecular changes processes and collectively advances our understanding of microbial processing of the marine elemental cycle.

**IMPORTANCE** Phytoplankton are a major contributor of labile dissolved organic matter (DOM) in the upper ocean, fueling tremendous marine prokaryotic activity. Interactions between microorganisms and algae-derived DOM regulate biogeochemical cycles in the ocean, but key aspects of their interactions remain poorly understood. Under global warming and eutrophication scenarios, *Synechococcus* blooms are commonly observed in coastal seawaters, and they significantly influence the elemental biogeochemistry cycling in eutrophic ecosystems. To understand the interactions between *Synechococcus*-derived DOM and heterotrophic prokaryotes as well as their influence on the coastal environment, we investigated the degradation of DOM by coastal microbes during a 180-day dark incubation. We showed substantial DOM compositional changes that were closely linked to the developments of microbial specialists and generalists. Our study provides information on the interactions between microbial population succession and DOM molecular changes, thereby advancing our understanding of microbial processing of the marine DOM pool under the influence of climate change.

## INTRODUCTION

The marine dissolved organic matter (DOM) pool contains ∼700 Pg C and represents one of the largest reduced carbon reservoirs on Earth (1, 2). Phytoplankton are a major source of organic matter in the upper ocean, releasing labile DOM to the surrounding environment that supports heterotrophic microbial activities (3, 4). In turn, the heterotrophic prokaryotes utilize labile DOM (LDOM) and, potentially, produce recalcitrant DOM (RDOM), which accumulates for millennia to become the dominant DOM pool in the ocean (5). The bioavailability of DOM is regulated by its intrinsic chemical molecular properties and molecular concentration in the marine ecosystem (5–8). In addition to the utilization and transformation of DOM, heterotrophic prokaryotes are also key players in other biogeochemical processes in the ocean, such as the elemental cycles of nitrogen, sulfur, and phosphorus (9, 10). Therefore, unraveling the interactions between phytoplankton-derived DOM and heterotrophic prokaryotes is essential to understanding the associations between the oceanic DOM pool and microbial-mediated cycling of carbon.

However, to date, few studies have addressed the molecular-level associations between DOM molecules and microbial communities in marine ecosystems (11–13). Elucidating the interaction of prokaryotic activities with DOM molecular composition has been challenging due to the tremendous chemical complexity of the DOM pool and the great diversity of microorganisms (14–17). Linking the information derived from organic geochemistry with molecular microbiology is a vital step in addressing the fundamental knowledge gap concerning the interaction between marine microorganisms and the DOM pool (11). Colored or chromophoric DOM (CDOM), including fluorescent DOM (FDOM), is an important fraction of DOM, and it has been observed in a wide range of aquatic environments (18). The optical properties of CDOM and FDOM can provide information about their origins and the potential bioavailability of DOM components without determination of molecular composition and structure (19). Further, Fourier transform ion cyclotron resonance mass spectrometry (FT-ICR MS) enables detailed molecular-level characterization regarding the composition of DOM, thereby allowing the assignment of molecular formulae (20–22). Although this technique cannot distinguish between specific molecular structures, it is a powerful tool for resolving the mass spectral composition of DOM in diverse environments (23, 24). Associating FT-ICR MS signal intensities of individual molecular formulae with FDOM components can offer important information about the bioavailability and source of DOM (16). With respect to prokaryotic communities, next-generation sequencing (NGS) of marker genes facilitates the classification of millions of reads of microbial DNA and RNA amplicons (11). The combination of DOM optical measurement, FT-ICR MS, and NGS approaches allows inference about the underlying pattern of microbial metabolism and fate of DOM in the oceans.

Cyanobacteria, consisting of mainly *Synechococcus* and *Prochlorococcus*, account for almost half of the primary production in oligotrophic oceans (25, 26). In addition, *Synechococcus* blooms, because of global warming and eutrophication, are now commonly observed in coastal seawaters, and they significantly influence the elemental biogeochemical cycling in eutrophic ecosystems (27, 28). For example, the dominant *Synechococcus* population was shifted from clade III to VIII in Florida Bay during bloom, and the latter could grow rapidly, utilize organic nutrients, and resist top-down control (29). However, the influence of *Synechococcus*-derived DOM on the coastal area environment during a bloom is poorly understood. In the previous study, we found close coupling between the different bioavailability of organic carbon (based on the turnover time of DOC concentrations) and the succession of microbial community structure in the *Synechococcus*-derived organic matter (SynOM) addition incubations (30). The bioavailability of DOM has been demonstrated to associate with its intrinsic chemical molecular properties and molecular concentration (7, 31). Moreover, the composition of microbial communities plays an important role in shaping the DOM molecular composition and vice versa (11, 32). Therefore, combined with our previous study (30), we hypothesized that the microbial-mediated SynOM organic molecular transformation, as well as its biological availability changes, was closely associated with bacterial community succession in the long-term incubations. To address this hypothesis, we followed the decomposition of SynOM by coastal prokaryotes using bioassay experiments. We traced the variation of total organic carbon (TOC) concentrations, FDOM components, and DOM molecular composition by ultrahigh-resolution FT-ICR MS, and compositions of the total and active prokaryotic communities were assessed by NGS of the 16S rRNA gene and rRNA, respectively, during a 180-day incubation experiment. The three main goals for this study were to (i) investigate the changes in molecular composition and bioavailability of *Synechococcus*-derived DOM by coastal microbes during the incubation, (ii) analyze the response and succession of the bacterial community along with the different forms of bioavailable organic matter, and (iii) explore the associations between microbial succession and DOM molecular changes.

## RESULTS

### Variation of total organic carbon and inorganic nutrients during the incubation experiment.

**FIG 1:**
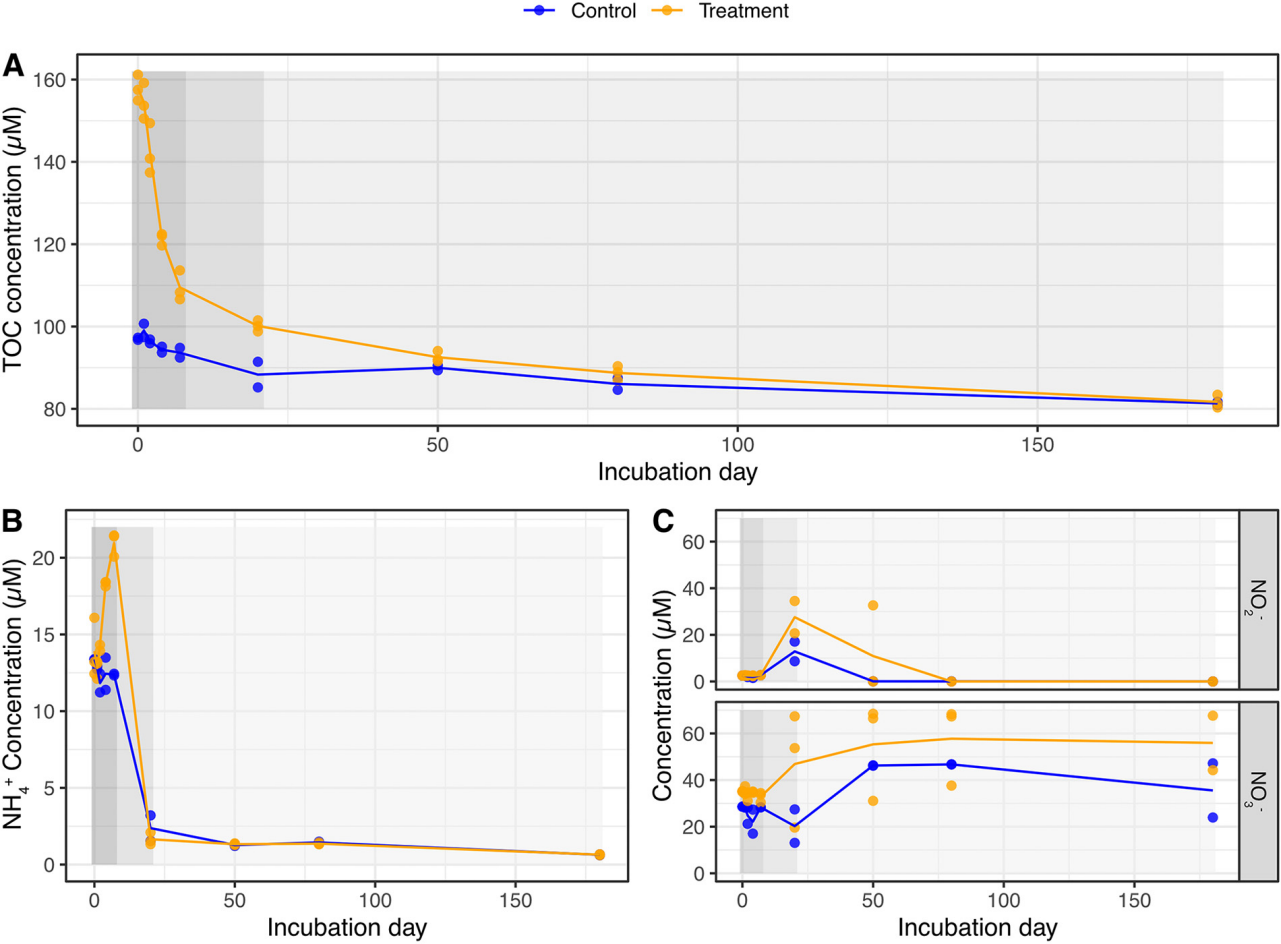
Dynamics of total organic carbon (A), ammonia (NH_4_^+^) (B), and nitrite and nitrate (NO_2_^−^, NO_3_^−^) (C) during the experiment. Orange and blue indicate the *Synechococcus*-derived DOM added sample and the control sample, respectively. The line indicates the average value of each attribute on each sampling day. Three phases (I, II, and III) characterized by TOC consumption rates are highlighted from dark gray to light gray. Phase I: days 0 to 7; phase II: days 8 to 50; phase III: days 51 to 180.

TOC concentrations in the SynOM amended microcosm incubations decreased from 158.0 ± 3.0 μmol C L^−1^ on day 0 to 82.0 ± 2.1 μmol C L^−1^ on day 180 (Fig. 1A). According to the utilization of organic matter in the treatment microcosms, the 180-day incubation period could be partitioned into three phases (I, II, III) using a multi-G model (33, 34), namely, phase I (days 0 to 7), phase II (days 8 to 20), and phase III (days 21 to 180) with TOC consumption rates of 7.0 ± 1.0, 1.0 ± 0.1, and 0.1 ± 0.0 μmol C L^−1 ^day^−1^, respectively. All three-phase models are statistically significant (all *P* values are <0.01). Compared to those in the treatment microcosms, the TOC concentrations in the controls were less variable, decreasing from 97.0 ± 0.0 to 81.0 ± 1.0 μmol C L^−1^ during the incubation.

The average concentration of nitrogen nutrients (e.g., NH_4_^+^, NO_2_^−^, and NO_3_^−^) was higher in the SynOM addition microcosms than in the control microcosms over the course of the experiment (Fig. 1B and C). Ammonia (NH_4_^+^) concentration in the treatment microcosms increased from day 0 (13.9 ± 0.1 μmol L^−1^) to day 7 (20.9 ± 0.8 μmol L^−1^) and then declined to a low concentration (1.6 ± 0.4 μmol L^−1^) by day 20. Correspondingly, NO_2_^−^ concentration increased between days 7 and 20, with the peak (29.3 ± 9.8 μmol L^−1^) on day 20, followed by a decline to the limit of detection on day 80 in the treatment microcosms. Subsequently, NO_3_^−^ concentration accumulated from day 20 (46.8 ± 24.6 μmol L^−1^) to day 80 (57.7 ± 17.4 μmol L^−1^) and then slightly decreased to 55.9 ± 16.5 μmol L^−1^ by the end of the experiment. These dynamic processes of inorganic nitrogen nutrients suggested that ammonia oxidization and nitrite oxidation occurred during the incubations. A similar pattern was observed for these three nutrients in the control microcosms but with a lower range of concentrations.

### Changes in optical properties of DOM during the incubations.

**FIG 2:**
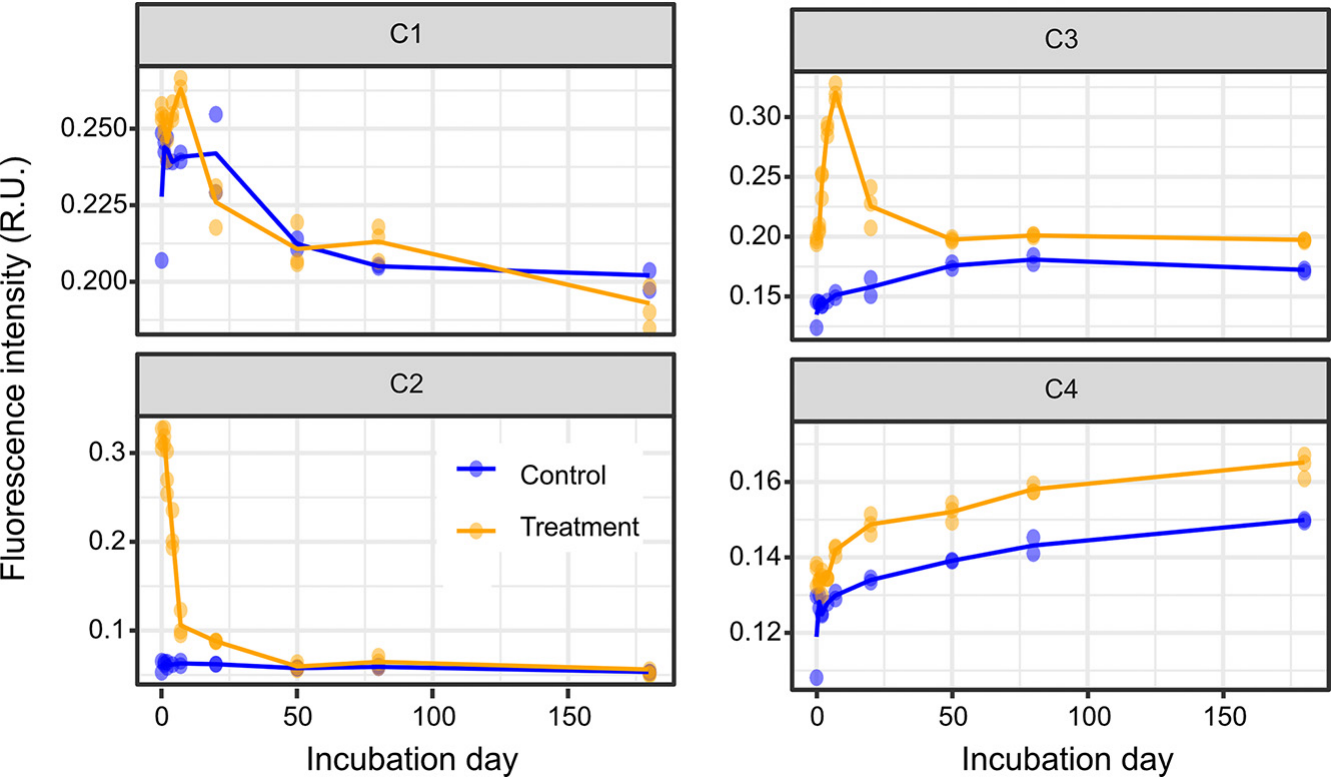
The intensity dynamic of component of fluorescence dissolved organic carbon defined by the PARAFAC model (C1 to C4). Blue and yellow dots represent each replicate, while the line represents the average intensity for control and *Synechococcus*-derived organic matter amendment microcosms, respectively.

Excitation-emission matrices (EEMs) and parallel factor analysis (PARAFAC) were integrated to evaluate changes in FDOM components during the experiment. Four FDOM components were identified: C1 (excitation [ex] | emission [em]: 240 | 352 nm), C2 ([255, 365] | 456 nm), C3 ([250, 340] | 404 nm), and C4 ([250, 385] | 484 nm) (Fig. 2A). The characteristics of the same components have been described in our previous studies (7, 30). Briefly, the C1 component exhibited a tryptophan-like peak, which was initially categorized as T peak (35). C2 component had a similar fluorescent property that was also detected in a culture of *Synechococcus* sp. CB0101 (36). C3 and C4 components were initially categorized as the M humic-like peaks M and C, respectively (35). The fluorescent intensity (expressed as Raman unit, R.U.) of the C1 component increased slightly during the first 7 days, from 0.25 to 0.26 (linear regression, *F*_1,13_ = 7.73, *P = *0.01) and 0.22 to 0.24 (linear regression, *F*_1,7_ = 0.27, *P = *0.66) in the treatments and controls, respectively, and then decreased stepwise during the experiment in the treatments (linear regression, *F*_1,13_ = 21.61, *P < *0.01) and in the controls (linear regression, *F*_1,8_ = 11.59, *P = *0.01) (Fig. 2B), suggesting its close association with microbial metabolic activities. The fluorescent intensity of the C2 component declined rapidly during the first 7 days (from 0.31 to 0.10, linear regression, *F*_1,13_ = 232.70, *P < *0.01), corresponding to the rapid utilization of organic matter in treatment microcosms and indicating labile reactivity. The fluorescent intensity of the C3 component continually increased during the first 7 days (from 0.19 to 0.32, linear regression, *F*_1,13_ = 201.90, *P < *0.01) and then declined rapidly from days 7 to 20 in treatment microcosms (from 0.32 to 0.21, linear regression, *F*_1,13_ = 4.37, *P = *0.05). This indicated that the C3 component was quickly produced by microorganisms when labile organic matter was abundant and was then utilized as the labile organic matter was consumed. These results suggested that the C2 and C3 components were directly or indirectly derived from the SynOM. Additionally, the fluorescent intensity of the C4 component increased by over 25% during the incubation in both treatment (from 0.13 to 0.17, linear regression, *F*_1,25_ = 145.50, *P < *0.01) and control (from 0.12 to 0.16, linear regression, *F*_1,15_ = 54.11, *P < *0.01), indicating its recalcitrant characteristic, and the fluorescent intensity in the treatment was greater than that in the control (average fluorescent intensity in treatment versus control: 0.15 ± 0.01 versus 0.13 ± 0.01, Welch’s *t* test, *t* = 3.10, df = 35.98, *P = *0.004). Based on their variation in fluorescent intensity, the components C2, C3, and C4 displayed labile, semilabile, and recalcitrant DOM characteristics, respectively.

### Changes in molecular signatures of SPE-DOM during the incubation.

In total, 5,654 molecular formulas (MFs) were detected and assigned across all the samples via FT-ICR MS, of which 1,766 were present in all the samples (Table S1). The number of MFs ranged from 2,932 to 3,799 overall samples, and MFs assigned to CHO ranged from 1,223 to 1,620, CHNO from 889 to 1,596, CHOS from 357 to 555, and CHNOS from 0 to 72. By relating the intensities of MFs with those of different bioavailable FDOM components (e.g., C2 and C4), we could predict the biological availabilities of MFs (16). Those MFs that had a significantly positive correlation with the recalcitrant C4 component (average *r* = 0.70 ± 0.1) showed a higher aromatic index (AI_mod_; 0.31 ± 0.13 versus 0.26 ± 0.18; Welch’s *t* test, *t* = 1.80, df = 92.36, *P = *0.07) and lower NOSC values (−0.41 ± 0.30 versus −0.04 ± 0.41; Welch’s *t* test, *t* = 5.60, df = 94.17, *P < *0.01) than MFs that were positively correlated with the labile C2 component (average *r* = 0.71 ± 0.1) (Fig. S1). This suggests that DOM molecular bioavailability is associated with aromaticity and redox state.

**FIG 3:**
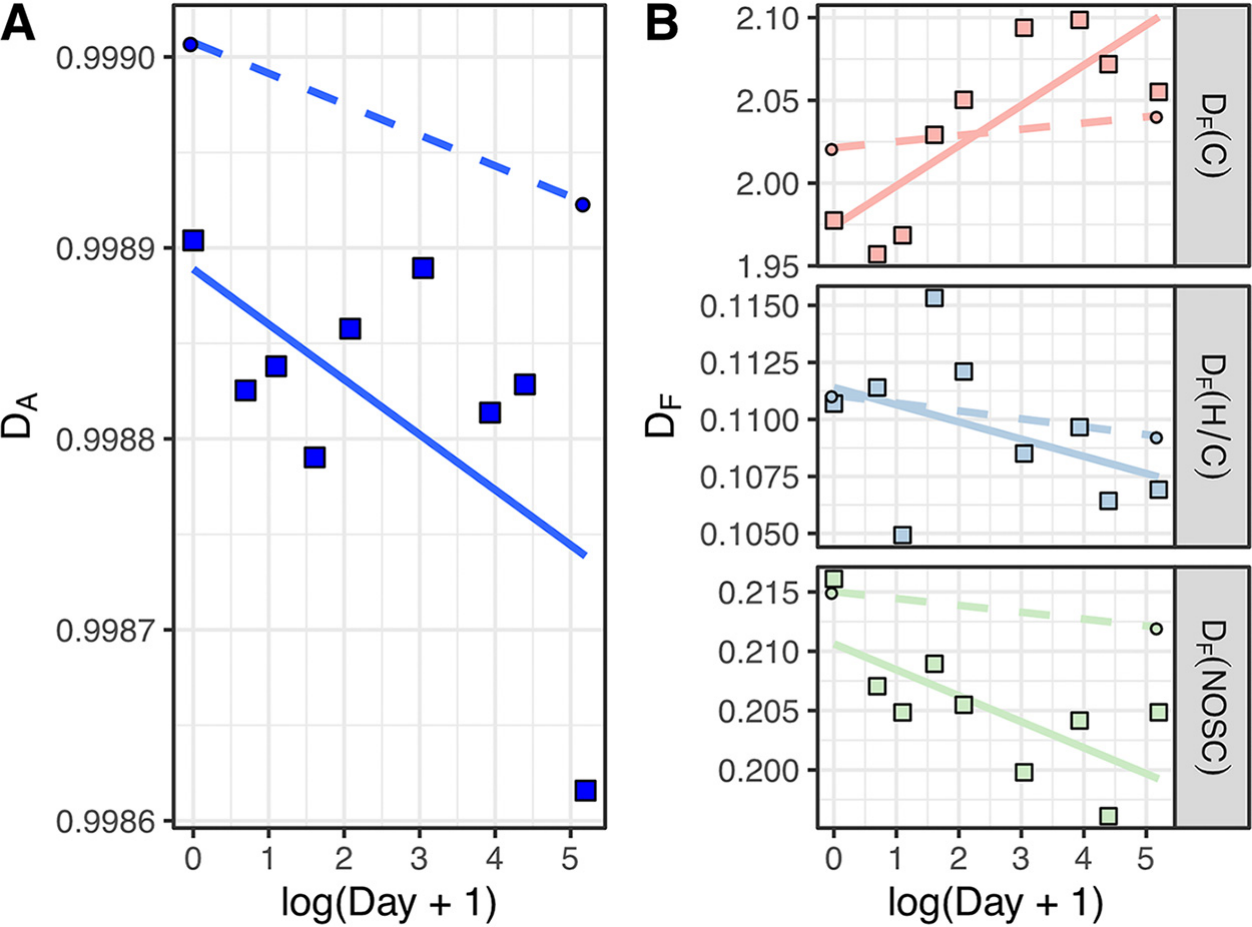
Abundance-based diversity (*D_A_*) and functional diversity (*D_F_*) of molecular formulae of DOM. *D_F_*(*C*), *D_F_*(*H*/*C*), and *D_F_*(*NOSC*) indicate the functional diversity based on the number of carbon atoms, H/C ratio, and NOSC, respectively. Squares indicate treatment samples, while dots indicate control samples. Solid lines indicate linear regression between log(day + 1) and diversity. Dashed lines show the trends of diversity changes from day 0 to day 180 in control, as only two samples were measured. The Pearson’s sum correlation significance *P* values for correlation between diversity and log(day + 1) in treatments are *D_A_*, *P* = 0.07; *D_F_*(*C*), *P* = 0.01; *D_F_*(*H*/*C*), *P* = 0.29; *D_F_*(*NOSC*), *P* = 0.04.

The MF diversity exhibited a significant change in the treatments during the experiment (Fig. 3). The abundance-based Gini-Simpson diversity index (*D_A_*) of DOM extracted using solid-phase extraction (SPE-DOM) was calculated based on the relative intensity of MFs, which describes the alpha diversity of SPE-DOM. The *D_A_* decreased in both the treatments and controls (Fig. 3A), suggesting that identified DOM molecular composition became more homogenous after microbial alteration. Furthermore, three structural properties were chosen to evaluate the changes in functional diversity of MFs, including carbon atom number [*D_F_*(*C*)], H/C ratio [*D_F_*(*H*/*C*)], and nominal oxidization state of carbon [*D_F_*(*NOSC*)] (Fig. 3B). The number of carbon atoms indicates the size and molecular weight of DOM molecules (within the analytical molecular window of FT-ICR MS), properties that are linked with their bioavailability (37), and the H/C ratio reflects the saturation of compounds: compounds with high H/C ratios are often degraded by bacteria more readily than compounds with low H/C ratios (38). The NOSC describes the average oxidation state of a molecule: the oxidation of an organic substance becomes, on average, thermodynamically more profitable as NOSC increases (39). The H/C ratio of DOM molecules partly reflects the NOSC value. Here, we observed an increase of *D_F_*(*C*) but decreases of *D_F_*(*NOSC*) in both treatment and control microcosms (Fig. 3B). Additionally, more MFs with a small (<15) or large (>20) number of carbon atoms were observed on day 180 than on day 0 in the treatment microcosms (Kolmogorov-Smirnov test for distribution of number of carbon atoms on day 180 compared to that on day 0, D = 0.04, *P = *0.01; Fig. S2).

### Composition of total (DNA-based) and active (RNA-based) microbial communities during the incubation.

**FIG 4:**
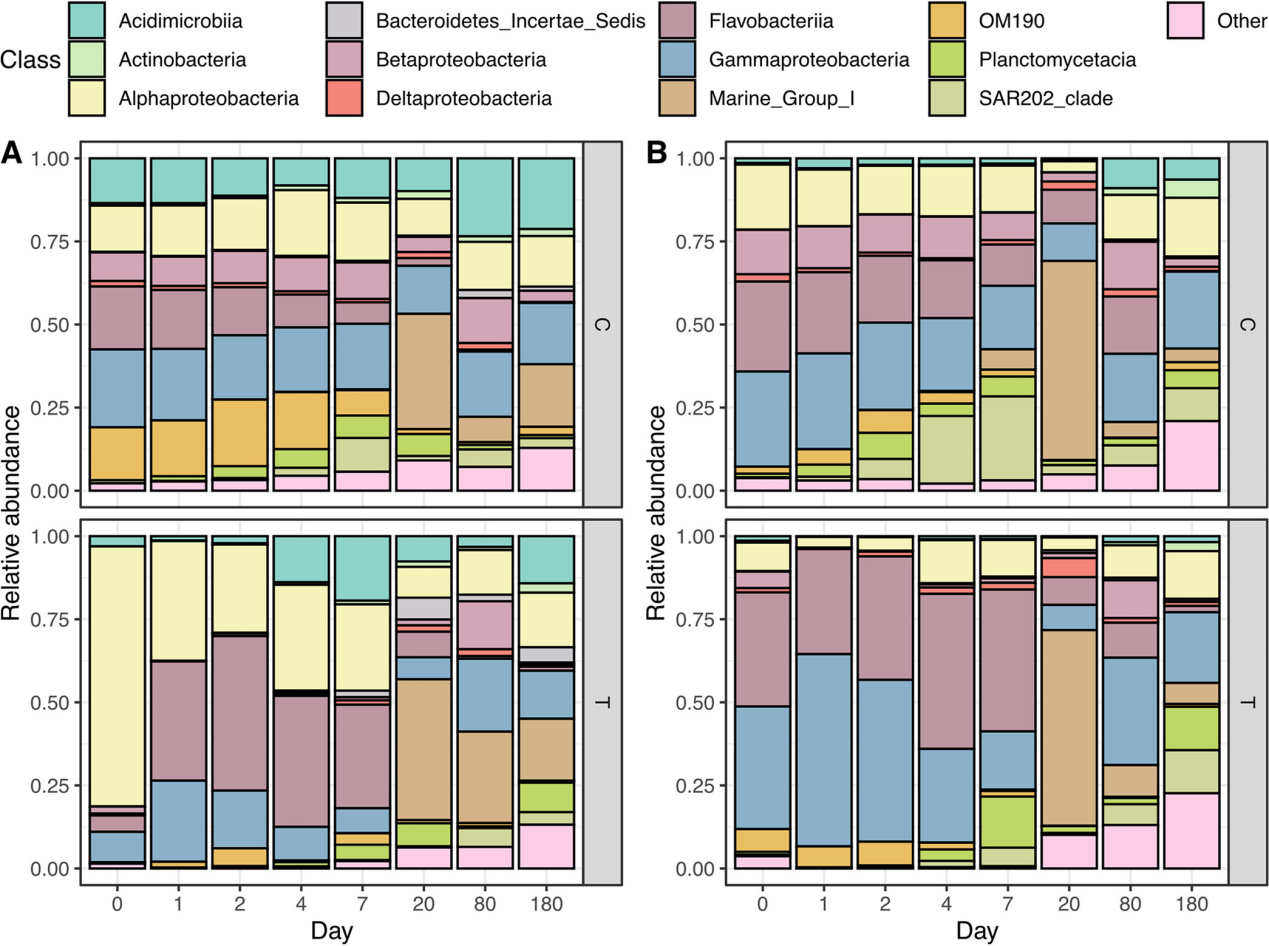
Structure of total (A) and active (B) microbial communities at the class level over the course of the incubation. T represents the microbial communities of the 0.22 to 3 μm size fraction in the treatment, while C represents the microbial communities of the >0.22 μm size fraction in the control. There was no addition of SynOM in the control, and seawater used in the incubation was prefiltered by 3-μm filters. Therefore, the structure of microbial communities in control microcosms had no size fractioning.

The microbial communities at the DNA level were characterized as the total microbial community including active, dead, or dormant microbes. In addition, microbial communities at the RNA level were the active microbial community, who could actively translate the rRNA genes. The Shannon index of the free-living microbial communities (0.22 to 3 μm size fraction) in treatment groups as well as that in control groups decreased for both active and total microbial communities over the course of the experiment (Table 1). Microbial community composition of the >3 μm size fraction obviously differed from that of the 0.22 to 3 μm fraction in treatment groups (details in the supplemental material, Fig. S3). In both treatment and control microcosms, the *Acidimicrobiia*, *Flavobacteriia*, SAR202 clade (within Chloroflexi), Marine Group I (MGI; *Thaumarchaeota*), *Planctomycetacia*, *Alphaproteobacteria*, and *Gammaproteobacteria* predominated over the course of the incubation (Fig. 4).

**TABLE 1:** Shannon index of microbial communities at DNA and RNA levels

Group	Shannon index of microbial communities at day:							
	0^*a*^	1	2	4	7	20	80	180
DNA								
Control	4.68	4.59	4.63	4.88	5.10	3.88	4.35	4.36
Treatment FL^*b*^	4.55	4.83	4.83	5.21	5.26	3.44	3.61	4.47
RNA								
Control	5.60	5.57	5.60	5.40	5.11	2.58	4.54	5.00
Treatment FL	5.41	5.39	5.40	5.82	4.70	2.47	4.85	4.84

a The experiment day.

b FL, indicates microbial communities are free-living (size fraction of <3 μm and >0.22 μm).

Additionally, we calculated a coarse class activity ratio (16S rRNA relative abundance/16S rRNA gene relative abundance at class level) to evaluate the activity of each class of bacteria. The result showed high activity of OM190, *Planctomycetacia*, *Betaproteobacteria*, and *Gammaproteobacteria* at the beginning of the experiment (Fig. 4 and Fig. S4). These bacterial groups were commonly detected microbial populations in sunlit coastal waters around Xiamen Island (40). It is notable that *Deltaproteobacteria* and the SAR202 clade showed high activity on day 4 followed by *Thaumarchaeota* on day 7. In contrast, *Actinobacteria* and *Alphaproteobacteria* were active during phase III of the experiment. Some rare species in the early phases became abundant and active in phase III, including *Acidobacteria*, *Actinobacteria*, *Chloroflexi* (major SAR202 clade), *Gemmatimonadetes*, and *Nitrospinae* (Fig. S5). These specific microbial taxa have been commonly reported as dominant populations in the deep sea or in surface sediment (41, 42). The microbial populations displayed a clear succession pattern from microbiomes dominant in surface seawater to those frequently detected in dark/deep ocean over the entire incubation period.

We constructed two structural equation models (SEMs) to evaluate the contribution of microbial community structure to the changes in bioavailability of DOM components (Fig. S6). In our models, we assumed that the utilization of labile DOM (LDOM; C2) and semilabile DOM (SLDOM; C3) by the microbes influenced the composition of microbial communities, further affecting the production of RDOM (C4 component), molecular composition, and diversity of SPE-DOM (details in the supplemental material). The SEMs results suggested that the active prokaryotes had a higher contribution to DOM changes than total prokaryotes. Therefore, we focused on active microbes in the following network analysis.

### Associations between DOM molecules and active microbial population.

**FIG 5:**
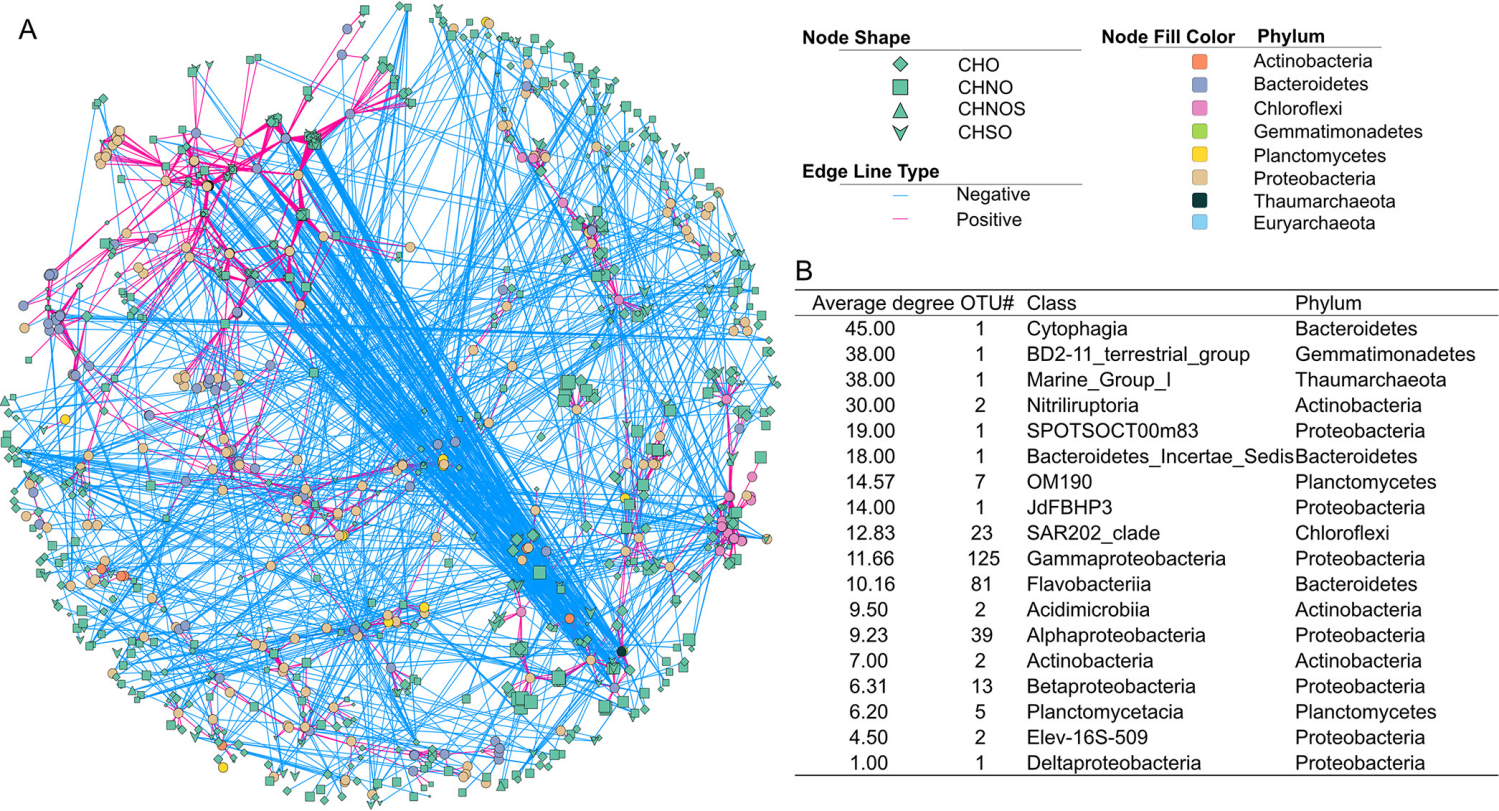
The large and complex subnetwork as based on the Spearman correlation between the active OTUs and MFs (A). Circles represent the active OTUs, while diamonds, squares, triangles, and arrows represent CHO, CHNO, CHNOS, and CHOS, respectively. The size of MFs indicates the *m/z* value. The dashed and solid edges indicate the negative and positive correlations with *P* values of <7.45e−05. Table B shows the average degree and OTU number of different active bacterial/archaeal classes in this subnetwork, where the higher degree of OTU indicates the more MFs it correlated with.

**FIG 6:**
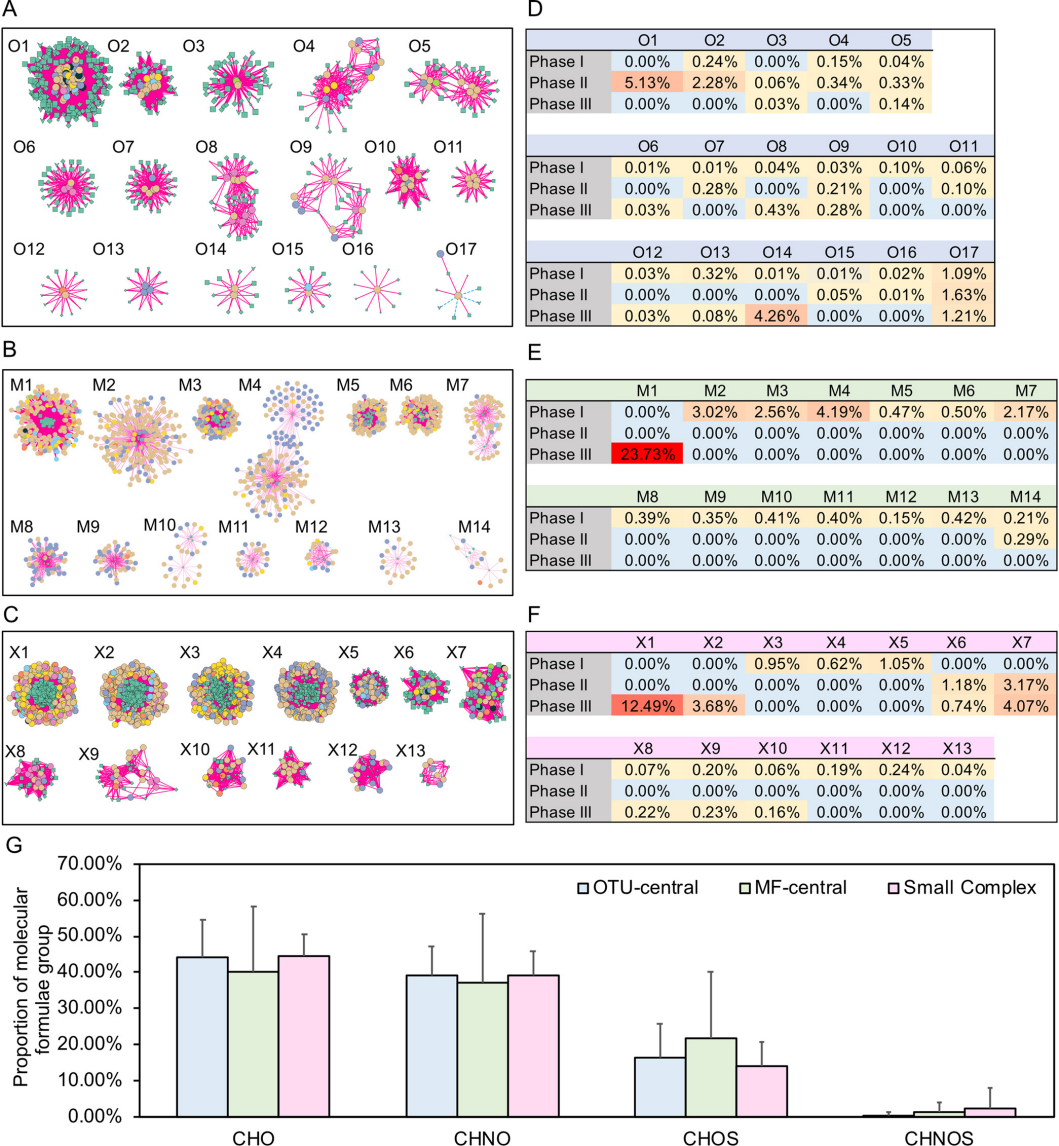
OTU-central subnetwork (A), MF-central subnetwork (B), and small and complex subnetworks (C). The legend of networks is the same as that in Fig. 5. Colors in panels D, E, and F indicate the average total proportion of OTUs in each subnetwork during different phases. Panel G represents the average proportion of molecular formulae groups of three types of subnetworks with standard deviations.

Marine DOM is a complex organic mixture that interacts with surrounding microbial species on a molecular level. Given this, networks based on the MFs and active operational taxonomic units (OTUs) were constructed to evaluate the relationships among the active microorganisms and organic molecules in our treatment incubations (Fig. 5 and 6). It is worth noting that the correlations do not necessarily indicate the actual degradation or production of DOM molecules by bacteria (43). In the network, the nodes represent DOM molecules or active OTUs, whereas the links between them represent significantly positive or negative correlations (blue or red, respectively). This network contained 2,886 OTUs and 2,484 MFs and was dominated by OTUs belonging to *Gammaproteobacteria* (36.76%), *Alphaproteobacteria* (14.00%), and *Flavobacteriia* (18.50%). All 2,484 MFs were assigned in the 14 DOM extracts. Their *m/z* values ranged from 221.0 to 514.1 (389.2 ± 68.5). CHO and CHNO were the major chemical molecular groups within the network (43.64% and 40.14%, respectively). A large and complex subnetwork was constructed (Fig. 5A) in which *Alphaproteobacteria* and *Gammaproteobacteria*, as well as *Flavobacteriia*, were dominant (details in the supplemental material).

In addition, a series of separated small subnetworks (Fig. 6A to C) were also formed. Here, 44 small, separated subnetworks containing more than nine nodes (a total of 84 subnetworks were found) were selected for further analysis. Different microbes correlated closely with distinct MFs within individual subnetworks (Fig. 6). For example, we found positive correlations between *Thaumarchaeota* and nitrogen-containing MFs (217 of total 516 positive correlations) as well as *Solimonadaceae* and MFs with large *m/z* values (438.3 ± 58.8 compared to 319.5 ± 40.4 of negatively correlated MFs) within the subnetworks. Furthermore, we found three types of subnetworks based on the fold value between OTU number and MF number (log_2_[fold]): OTU-central (log_2_[fold] ≤ −1, e.g., Fig. 6A), MF-central subnetwork (log_2_[fold] ≥ 1, e.g., Fig. 6B), and other complex subnetworks (−1 < log_2_[fold] < 1, e.g., Fig. 6C). OTU-central subnetworks contained active microbial populations that displayed high relative abundance during all the incubation phases, while MF-central subnetworks contained the active microbes with high relative abundance during incubation phase I, except for subnetwork M1 (Fig. 6D to F and Table S2). Additionally, the MF-central subnetworks consisted of lower average proportions of CHO and CHNO but a higher proportion of CHOS compared to OTU-central and complex subnetworks (Fig. 6G and Table S3). These results suggested that the succession of prokaryotic communities covaried with different bioavailable DOM molecules over the course of the experiment.

## DISCUSSION

### Changes in DOM bioavailability, molecular signature, and diversity mediated by microbial degradation.

*Synechococcus*-derived OM (SynOM) contains a large amount of biologically available organic matter. Over the 180-day incubation, 76.2 μmol C L^−1^ TOC was utilized in the SynOM addition microcosms and only 15.7 μmol C L^−1^ in the controls, suggesting that most SynOM was utilized. Considering that ∼60 μmol C L^−1^ SynOM was added in the treatment, there was no clear priming effect observed over the entire experiment (44).

The optical properties of FDOM are promising tools for studying their potential bioavailability (7). According to variation in fluorescence intensity, labile (C2), semilabile (C3), and recalcitrant (C4) FDOM components were identified in our incubation (Fig. 2A and B). The fluorescence intensity of the C1 component was similar between control and treatment microcosms, suggesting that the C1 component was autochthonous LDOM in this eutrophic coastal seawater. This component showed a tryptophan-like fluorophore that was initially categorized as a T peak (35). In contrast, the obviously higher fluorescence intensity of the C2 and C3 components in the treatment indicated that they were primarily extrinsic from the SynOM addition. The C2 component showed a similar fluorescent signal that was detected in a 15-day culture of *Synechococcus* sp. CB0101 (36). The C3 component was initially characterized as the M humic-like peak (35). These results suggested that *Synechococcus*-derived DOM might comprise two major labile FDOM components for the heterotrophic bacterial utilization. In contrast, the fluorescence intensity of the C4 component increased over the entire incubation period in both control and treatment microcosms, indicating that it was resistant to microbial degradation. *Synechococcus* pigments were previously reported to significantly contribute to the production of the C4 component (36). In the context of the specific microcosm system, the C4 component thus represents a class of potential RDOM molecules that are also widely distributed in the open ocean water column (45). Higher fluorescence intensity of the C4 component in the treatment suggests that the addition of SynOM increased potential FDOM component accumulation in the closed microcosm system.

Although the nutrient condition of collected seawater in the present study (station S07) was different from that in our previous investigation (stations S03 and S05), the variable patterns of FDOM components and TOC and inorganic nutrient concentrations were similar, as well as the succession of microbial community structure (30). In the present study, we focused mainly on the interactions between microorganisms and DOM at the molecular level by linking the microbial population succession and DOM molecular changes over the entire incubation period.

The addition of 60 μmol C L^−1^ SynOM, primarily comprising labile organic matter, greatly influenced the DOM composition and fueled microbial activities in the closed incubation system. The results of this study support the hypothesis that the interactions between active microbial composition and DOM composition are driven by “patchy,” short-lived events such as phytoplankton blooms (11). The active microbial composition was more influenced by labile or semilabile DOM (e.g., C2 and C3 components) and played important roles in shaping DOM molecular composition and RDOM production (C4 component) in our incubation experiments (Fig. S6).

The bioavailability of DOM is related to its intrinsic chemical molecular properties (7, 31, 46). We found that DOM molecules with lower AI_mod_ and higher NOSC were more easily degraded by prokaryotes (Fig. S1). High-aromaticity DOM molecules have been shown to require more energy to be degraded by microorganisms (47, 48). Consistent with this, microbial degradation results in DOM along a gradient from a high to low NOSC in the aquatic environment (16). Therefore, our results demonstrate that the degradation by microorganisms increased the aromaticity but decreased the redox state of DOM produced by *Synechococcus*, which consequently affected the change in composition and chemodiversity of the DOM pool.

The chemodiversity of SPE-DOM, including abundance-based diversity and functional diversity except for *D_F_*(*C*), declined in both treatment and control microcosms (Fig. 3). These results suggested that the molecular composition of the original DOM and *Synechococcus-*derived DOM became more homogenous and less bioavailable over time in our closed microcosm system. Similarly, it was found that viral-induced DOM from *Synechococcus* cells was converted to a relatively stable DOM pool after microbial processing (13). This is consistent with a previous field study that found increased homogeneity of chemical properties in the more degraded DOM (49).

### DOM composition impacts the populations of specialists and generalists.

DOM is usually considered the prevailing energy and carbon source for the largely heterotrophic prokaryotes, and its composition is closely related to the microbial metabolic characteristics (50). The composition and bioavailability of DOM have a great influence on the composition of microbial communities in the coastal ocean (32). In line with the previous study (30), the high relative abundance of *Alphaproteobacteria*, *Gammaproteobacteria*, *Bacteroidetes*, and *Actinobacteria* was found during phase I due to their quick metabolic response to the LDOM (51–54). During phase II, SLDOM was consumed after most LDOM was depleted in phase I. In this phase, *Thaumarchaeota* was the dominant group and drove the transformation of nitrogen nutrients from ammonia into nitrite (30). A slightly higher proportion of N-containing DOM MFs was also found on day 20 (Table S1), which suggests that inorganic and organic nitrogen were closed coupling. In phase III, most biological available organic matter was used up and RDOM was accumulated continuously. The *Planctomycetales*, SAR202 clade, *Alphaproteobacteria*, and *Gammaproteobacteria* became the dominant groups, which indicates that some bacterial groups within these classes have the ability to utilize the relatively recalcitrant DOM (51–54).

It has been proposed that bacterial metabolism properties (i.e., specialists and generalists) and DOM properties (i.e., bioavailability) determine how communities respond to DOM heterogeneity (50). Decreased microbial community diversity along with DOM chemodiversity suggested that the loss of chemical “niche” or substrates resulted in fewer opportunities for microbial mineralization of DOM. Specialists are sensitive to environmental changes in their specific substrate preferences (11, 55, 56). Here, we found that the active bacteria within MF-central subnetworks were present at relatively high abundance during phase I or II but nearly disappeared during phase III, indicating that the limited bioavailable DOM restricted these specialists (Fig. 6D to F and Table S2). Compared with those in other MF-central subnetworks, OTUs within subnetwork M1 showed a high relative abundance during phase III (Fig. 6E), suggesting that they may benefit from the conditions of low labile DOM concentration during this phase. Likewise, OTUs within subnetworks X1 and X2 also showed a high relative abundance during phase III (Fig. 6F). Although X1 and X2 were characterized as complex subnetworks under our cutoff, we found more MFs than OTUs in these subnetworks. These two subnetworks were dominated by *Xanthomonadales* (especially genus *Polycyclovorans*), SAR202 clade, *Rhodospirillaceae*, and *Planctomycetales* (especially genus *Planctomyces*), all of which have been characterized as specialists (51–54). Additionally, these bacteria are prevalent in the deep ocean (57) where RDOM is abundant (31).

In contrast, generalists were present during almost all three phases but at low relative abundances (Fig. 6), indicating their resistance to environmental changes (55, 56). Resource generalists have been demonstrated to dominate coastal bacterial communities (58) and are common in aquatic environments (50). The utilization of multiple DOM molecules by generalists, which requires the production of extra enzymes and transporters, is costly compared to that by specialists (59). It has been proposed that generalist, multivorous bacteria may actually have a fitness advantage if resources are interchangeable (60). Therefore, our results implied that bacterial communities tend to be composed of a few abundant specialists and a diverse group of generalists during the transformation of DOM molecules from labile to recalcitrant. This is consistent with a previous model simulation showing that the marine DOM pool tends to be more functionally recalcitrant driven by diverse generalists (61). Since each member of marine microorganism plays essential roles in metabolizing *in situ* organic matter, the changes in diversity and connectivity of specialists and generalists might affect the stability of the marine DOC pool (62).

### Conclusions.

**FIG 7:**
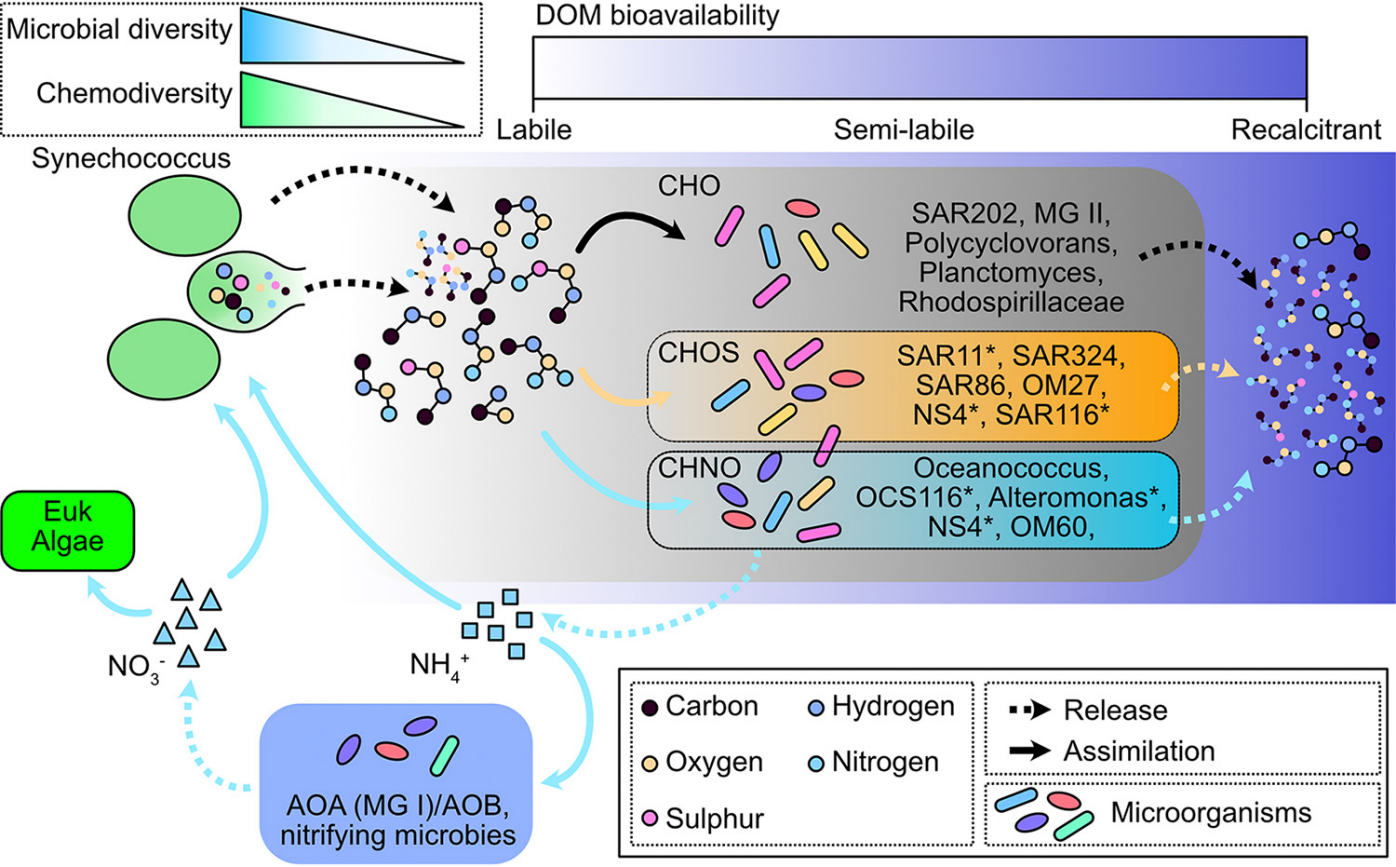
Schematic figure showing the *Synechococcus*-derived DOM transformation by the bacteria and archaea. Molecular weight of the DOM is represented by the cycle size. The bioavailability of DOM from labile to recalcitrant is indicated by the color bars from white to dark blue. The dark blue squares highlight the nitrification involved in ammonia oxidization archaea/bacteria (AOA/AOB) and other nitrifying bacteria. Solid arrows indicate assimilation of organic matter or inorganic nutrients by archaea/bacteria, while the dashed arrows indicate the release of organic or inorganic matter by microorganisms. The representative bacteria or archaea are shown in the squares, where asterisks indicate the bacteria/archaea involved in the metabolism of CHOS and CHNO. Colored circles indicate the different atoms, where the size indicates the *m/z* value. Dashed squares show the trend of microbial diversity and chemodiversity of DOM during the experiment.

In this study, we investigated the changes of SynOM by coastal microorganisms over a 180-day dark incubation. The experiment was partitioned into three phases based on the TOC consumption rates in SynOM addition groups. The identified FDOM components displayed different reactivities, from labile to recalcitrant properties. Furthermore, the DOM molecules exhibited increased *D_F_*(*C*) and decreased *D_F_*(*H*/*C*) and *D_F_*(*NOSC*) values over the course of the incubation, suggesting changes in organic molecular functional diversity. In addition, microbial populations were dominated by groups commonly found in the sunlit surface seawater in the initial experiments, and they shifted to those frequently detected in dark/deep ocean at the end of the 180-day dark incubation. Overall, the covariation of DOM molecules and microbial populations sheds light on their molecular-level interactions (Fig. 7) and expands our understanding of microbial-mediated carbon cycling in the ocean. Climate changes, including decreasing pH, increasing temperature, and coastal eutrophication, greatly influence the distribution and growth of phytoplankton and stimulate *Synechococcus* blooms in the coastal regions. Thus, the increased amount of organic matter produced by *Synechococcus* might alter the marine DOM pool and cause changes in ecosystem function in the oceans.

## MATERIALS AND METHODS

### Experiment setup and sampling.

The experimental setup has been described previously; the study was performed at stations S03 and S05 (30). Briefly, *Synechococcus* sp. XM-24 isolated from coastal waters near Xiamen Island, China (63) was grown in liquid SN medium at 22°C and harvested during the exponential growth phase (∼15 days, ∼1 × 10^7^ cells/mL) to obtain *Synechococcus*-derived organic matter (SynOM). *Synechococcus* cells were collected by centrifugation and subjected to multiple freeze-thaw cycles to extract SynOM (64). The collected SynOM was stored at −20°C until the experiment start. A total of ∼50 L surface seawater at depth of 5 m was collected from station S07 in the coastal region near Xiamen Island, China (24.50° N, 118.24° E) on 29 June 2017. Seawater from S07 is influenced by saline water from the South China Sea (40) and shows higher salinity and lower nutrient levels than S03 and S05, which were investigated in our previous study (30). The experiment was carried out in 10 l-polycarbonate bottles that were pre-acid-washed and rinsed with sample water. Each microcosm (*n* = 5; SynOM was added to three microcosms as treatment, while no SynOM was added in the other two microcosms as control) was established with 10 L of seawater that was prefiltered through a 3-μm filter for removing microeukaryotes and particulates (Millipore, Bedford, MA, USA). The filter system was precleaned with ultrapure water to minimize carbon contamination.

*Synechococcus* blooms are commonly observed in coastal seawaters, and the highest abundance reported to date was found in the Comacchio lagoon system (NW Adriatic coast), up to 2.4 × 10^7^ cells mL^−1^ (65), which is approximately equal to 50 to 60 μmol C L^−1^, assuming 20 fg C cell^−1^ (66, 67). In this study, we amplified *Synechococcus* sp. contribution approximated to local primary production to simulate the microbial response to pulse increases of organic matter from possible *Synechococcus* blooms occurring in the coastal regions in the future. Therefore, treatment groups (*n* = 3) were amended with SynOM of ca. 60 μmol C L^−1^; two carboys without SynOM additions were used as control groups.

Microcosms were incubated at 26 ± 0.5°C in the dark for 180 days without mixing (except for sampling). During the experiment, a filter with 0.22-μm pore size was installed on the microcosm to avoid environmental microbial contamination and keep air exchange. Subsamples were taken on days 0, 1, 2, 4, 7, 20, 50, 80, and 180 and stored at −20°C until analysis. Total organic carbon (TOC), dissolved organic carbon (DOC), and fluorescent DOM (FDOM) samples were collected. In addition, 300 mL of water samples in control carboys were filtered through 0.2-μm polycarbonate membrane filters (47 mm diameter, Millipore, USA) for DNA and RNA analysis. In addition, 300 mL of water in treatment groups was filtered through 3- and 0.2-μm polycarbonate membrane filters (>3 μm and 0.22 to 3 μm size fractions). Details of sampling for TOC, DOC, and microbial community composition were shown in the supporting information.

### Analysis of TOC, DOC, and dissolved inorganic nitrogen nutrient concentrations.

TOC and DOC concentrations were measured by high temperature (680°C) catalytic oxidation (HTCO) using a Shimadzu TOC-VCPH analyzer (Shimadzu, Kyoto, Japan) (68). Samples were defrosted and acidified to pH 2 with phosphoric acid. Then, the samples were standardized using potassium hydrogen phthalate and all measurements were quality controlled using Consensus Reference Material (Hansell Organic Biogeochemistry Laboratory, University of Miami, FL, USA) (2). Concentrations of dissolved inorganic nutrients, including nitrite (NO_2_^−^) and nitrate (NO_3_^−^), were measured via spectrophotometric methods (69) using a Technicon AA3 auto analyzer (Bran+Luebbe GmbH, Hamburg, Germany). Indophenol blue (IPB) spectrophotometric methods were used to analyze the ammonium (NH_4_^+^) concentration in samples (70). However, it is notable that the concentrations of DOC were slightly higher than those of TOC after day 7 in treatments, indicating the possible introduction of extra DOC by sampling.

### DNA and RNA extraction and sequencing.

The DNA and RNA extraction procedures have been described previously (30). Briefly, genomic DNA was extracted by the phenol, chloroform, and isoamyl alcohol method, followed by quality measurement using a NanoDrop ND-1000 spectrophotometer (Thermo Fisher Scientific, Waltham, MA, USA). RNA was extracted using the TRIzol method (Invitrogen, Waltham, MA, USA), and cDNA was generated using a SuperScript first strand synthesis system with random primers followed by synthesis of the second-strand cDNA using RNase H and DNA polymerase I (Invitrogen, Waltham, MA, USA). DNA and cDNA amplification of the bacterial 16S rRNA genes (V4–V5 region) was performed using the forward primer 515F (5′-GTGCCAGCMGCCGCGGTAA-3′) and the reverse primer 907R (5′-CCGTCAATTCMTTTRAGTTT-3′) (30). Sequencing libraries were generated using an NEB Next Ultra DNA library prep kit (New England Biolabs, Waltham, MA, USA) for Illumina following the manufacturer’s recommendations. The library was sequenced on an Illumina MiSeq platform (Shanghai Personal Biotechnology Co., Ltd., Shanghai, China), generating 450 bp paired-end reads. Detailed quality control of sequences is described in the supplemental material. Operational taxonomic units (OTUs) were clustered based on high-quality sequences (Q value of ≥Q20 and length of ≥150 bp) with a 97% similarity cutoff using UPARSE (v7.0.1001). The OTUs were taxonomically classified based on the SILVA database (version 132).

### Fluorescence and PARAFAC modeling.

Excitation-emission matrices (EEMs) were collected on filtered seawater (0.45 μm mixed cellulose ester membranes). Fluorescence measurements were performed using a 1-cm quartz cuvette and a Varian Cary Eclipse spectrofluorometer (USA). Emission spectra were scanned every 2 nm at wavelengths from 280 to 600 nm, with excitation wavelengths ranging from 240 to 450 nm at 5-nm intervals. Slit widths were 10 nm for both excitation (ex) and emission (em). Milli-Q water was used to generate the blank EEMs. Inner filter effect and lamp corrections were conducted. The EEMs and blank EEMs were scanned on the same day, and sample EEMs were blank subtracted and Raman normalized (71). EEMs were decomposed into components using parallel factor analysis (PARAFAC) using MATLAB 2012 and the DOMFluor toolbox (19). It is notable that the FDOM samples were frozen before measuring, which might affect the spectral properties of DOM (72, 73). However, all the samples were treated under the same condition, and the results still reflected the comparable changes over the incubation as well as the difference between treatment and control.

### SPE-DOM and FT-ICR MS analysis.

Filtered water samples from each replicate were pooled and then acidified to pH 2 with HCl. DOM was extracted using solid-phase extraction (SPE-DOM) by hydrophobic styrene-divinyl-polymer type PPL cartridges (Bond Elut PPL, 200 mg, 3 mL, Agilent, USA) following methods described elsewhere (74, 75). FT-ICR MS measurement on SPE-DOM was employed as described in a previous study (7). SPE mass peaks with a signal-to-noise ratio of 4 or higher were kept for formulae assignment, and formulae were analyzed over a range of 225 to 850 Da. Relative intensities, double bond equivalents (DBE), modified aromatic indices (AI_mod_), nominal oxidation state of carbon (NOSC), H/C ratios, and O/C ratios were calculated for each sample as described elsewhere (7). We calculated AI_mod_ and NOSC of each MF as described in previous studies (16, 47, 48):

$$AI_{\mathrm{mod}}=\frac{1\;+\;C\;–\;0.5O\;–\;S\;–\;0.5N\;–\;0.5H}{C\;–\;0.5O\;–\;S\;–\;N}$$

AI_mod_ values 0.5 to 0.67 and > 0.67 were assigned as aromatic and condensed aromatic structures, respectively.

$$\mathrm{NOSC}=4\;-\;\frac{4C\;+\;H\;-\;3N\;-\;2O\;-\;2S}{C}$$

where C, H, N, O, and S refer to the number of atoms per formula of carbon, hydrogen, nitrogen, oxygen, and sulfur, respectively.

To evaluate the change in the diversity of molecular formulae, two diversity indices were calculated in our study, an abundance-based Gini-Simpson index (*D_A_*) and a functional diversity index, Rao’s entropy *D_F_*(*C*) (49). The *D_A_* was calculated as follows:

$$D_{A}=1\;-\overset{N}{\underset{i\;=\;1}{\sum }}p_{i}^{2}$$

where *N* is the total number of molecular formulas in the data set and *p_i_* is the relative signal intensity of the MF *i*. The value *D_A_* indicates the probability that the MFs of two randomly chosen molecules (selection with replacement) differ.

The *D_F_*(*C*) was calculated as follows:

$$D_{F}\left(C\right)=\overset{N-1}{\underset{i=1}{\sum }}\overset{N}{\underset{j=i+1}{\sum }}p_{i}\times p_{j}\times \left|c_{i}-c_{j}\right|$$

where *c_i_* is a quantitative chemical property of the MF *i* (e.g., *m/z* or number of carbon atoms). For all molecule pairs (*i*, *j*) in the sample, the absolute difference in this property, $|c_{i}-c_{j}|$, is weighted by the sum-normalized signal intensities *p_i_* and *p_j_* of the two molecules. The value *D_F_*(*C*) indicates the expected difference between two molecules with respect to the chosen property. The selection is with replacement. Additionally, it was assumed that the relative signal intensity represents the abundance of molecules.

It is notable that both optical measurement and FT-ICR MS techniques have specific analytical windows, and thus different fractions of DOM are captured. Optical measurements consider only the colored/fluorescent pools of DOM. Furthermore, FDOM can be affected by quenching processes that depend on the sample matrix (76). The FT-ICR MS approach, however, excludes very small ionic compounds and larger colloidal aggregates due to solid-phase extraction and electrospray ionization (ESI) (20, 77). Therefore, some large and small DOM molecules were not captured in our study. SPE extraction efficiency estimated from organic carbon concentration was ∼40% (74). It should be noticed that DOM molecular composition was measured at only two time points (days 0 and 180) in the control. In addition, the SynOM obtained by freeze-thaw cycles might induce highly labile organic matter (e.g., ATP, DNA, or RNA) compared to natural exudation, viral lysis, or predation, which might affect the microbial metabolizing DOM processes. However, we found similar variable trends of the chemical diversity and molecular properties of DOM (e.g., *m/z* and AI_mod_) in control and treatment incubations (Fig. 3 and Table S1).

### Statistical analysis.

Structural equation models (SEMs) were employed to infer the relative importance of the C2 component, C3 component, MF diversity (*D_A_*) and composition, and bacterial composition on the C4 component by the *piecewiseSEM* package in R (78). Piecewise SEM can (i) piece multiple separate (generalized) linear models together into a single causal network, (ii) use Shipley’s test of d-separation to test whether any paths are missing from the model, and (iii) use the Akaike information criterion (AIC) to compare nested models and the effect of small sample sizes (78). We constructed two piecewise SEMs. One assumed that active bacterial composition drives the MF diversity and composition as well as the RDOM pool; the other assumed that total bacterial composition was the driver. We fitted the component models of the piecewise SEM as linear models. FDOM component, bacterial diversity and composition, and DOM diversity and composition were log-transformed. The overall fit of the piecewise SEM was evaluated by Shipley’s test of d-separation, Fisher’s C statistic, and the AIC (78).

The correlations between the MFs and FDOM and between MFs and nutrients were calculated, and the correlations with *P* values of <0.01 were retained. In addition, the correlation among MFs and OTUs was corrected through the false-discovery rate method (79, 80), and then associations with a *P* value of <7.45e−05 were kept for further network construction by igraph. The networks were visualized via Cytoscape 3.7.0.

The normality of the data was tested by the Shapiro-Wilk normality test. If the distribution of data was not normal, data were log-transformed to match the normal distribution. All the statistical analyses were conducted in R (version 3.6.0).

### Data availability.

Sequence data were deposited in the National Center for Biotechnology Information (NCBI) Sequence Read Archive with BioProject PRJNA623552. FT-ICR MS data were deposited on figshare with doi.org/10.6084/m9.figshare.12369236.v1.
