# Supplementary material for: Linking Microbial Population Succession and DOM Molecular Changes in Synechococcus-Derived Organic Matter Addition Incubation
Source: Microbiol Spectr. 2022 Apr 5;10(2):e02308-21. doi: 10.1128/spectrum.02308-21 (PMC9045170; doi:10.1128/spectrum.02308-21)
Supplement: SUPPLEMENTAL FILE 1 — Supplemental material. Download SPECTRUM02308-21_Supp_1_seq11.pdf, PDF file, 3.0 MB [file spectrum02308-21_supp_1_seq11.pdf]

Supporting information for Linking microbial population succession and DOM molecular changes in *Synechococcus*-derived organic matter addition incubation

Yu Wang[1,2], Rui Xie[1,2], Yuan Shen[1], Ruanhong Cai[1,2], Chen He[3], Qi Chen[1,2], Weidong Guo[1,4], Quan Shi[3], Nianzhi Jiao[1,2] and Qiang Zheng[1,2#]

[1]State Key Laboratory for Marine Environmental Science, Institute of Marine Microbes and Ecospheres, College of Ocean and Earth Sciences, College of the Environment and Ecology, Xiamen University, Xiamen 361102, China.

[2]Fujian Key Laboratory of Marine Carbon Sequestration, Xiamen University, Xiamen 361102, China.

[3]State Key Laboratory of Heavy Oil Processing, China University of Petroleum, Beijing 102249, China.

[4]Key Laboratory of Coastal and Wetland Ecosystems, Ministry of Education, Xiamen University, Xiamen 361102, China.

Sampling for TOC, DOC, DNA and RNA

For total organic carbon (TOC) measurement, 20 mL of water samples were collected directly into 40 mL glass vials (CNW, Germany) and immediately stored at -20°C until further analysis. For dissolved organic carbon (DOC) and fluorescent DOM (FDOM) measurements, 20 mL of water samples were filtered through pre-combusted (450°C, 4 h) 0.7μm pore-size GF/F filters (47 mm diameter, Whatman, Maidstone, UK) into 40 mL glass vials and stored at -20°C. Additionally, a total of 200 mL sample collected in duplicate and triplicate from controls and treatments, respectively, was filtered by pre-combusted GF/F filters in an acid-cleaned glass bottle under positive pressure for further solid-phase extraction. To prevent carbon contamination, all glass materials used for sample collection and storage were acid-washed, Milli-Q (ion- and nuclease-free water) rinsed, and combusted at 450°C for 4 h.

For DNA and RNA analysis, 300 mL of water samples were immediately filtered through 0.2 μm polycarbonate membrane filters (47 mm diameter, Millipore, USA). Since the particles from SOM be added in treatment microcosms, size fractionation may play more important roles in the treatment microcosms. For treatment groups, 300 mL of water was filtered through 3 and 0.2 μm polycarbonate membrane filters (>3 μm and 0.22 to 3 μm size fractions). Samples for RNA extraction were collected within 30 min and stored in 2-mL RNase-free tubes with RNA stabilization solution (Ambion, USA). All filters were flash-frozen in liquid nitrogen for 10 min and subsequently stored at -80°C until DNA or RNA extraction.

Phase separation using multi-G model based on TOC cenctration

We use multi-G model to confirm our organic matter degradation stage (Guillemette and del Giorgio, 2011; Jørgensen, 1978):

$$TOC(t) = TOC(Lab)[\exp(-kt)] + TOC(Res)$$

where TOC is the total TOC concentration at the beginning of the experiment; TOC(Lab) and TOC(Res) are the labile and the residual pools of estimated by model, respectively. In comparison with original formula, we used TOC instead of the DOC since the DOC were not available and comprised small proportion of organic carbon in our experiment. We first tested our phase separation. All the models are statistically significant (all p values < 0.01). The results indicated the k decreased from 0.160 to 0.001 (phase I: 0.160; phase II: 0.052; phase III: 0.005).

Variations of inorganic phosphorus

We also measured the PO_4^{3-} concentration along the experiment. However, a small fluctuation in PO_4^{3-} concentration was detected in both groups of microcosms, ranging from 1.608 ± 0.069 to 2.420 ± 0.042 μmol L^{-1} in the treatment and from 1.174 ± 0.045 to 1.637 ± 0.008 μmol L^{-1} in the control.

SPE-DOM and FT-ICR MS analysis

We used solid phase extraction (SPE) to collect the DOM. After extraction of DOM, the cartridges were rinsed with 6 mL of ultrapure acidified water (pH 2) to remove residual salts, then dried with high purity N_2. Finally, SPE-DOM was eluted by 2 mL

of high-pressure liquid chromatography grade methanol (Sigma-Aldrich, Germany). The complex molecular character of SPE-DOM was subsequently analyzed using a 9.4 T Bruker Apex ultrahigh-resolution FT-ICR MS with an Apollo II electrospray ion source operated in negative mode. SPE-DOM samples in methanol were injected into the electrospray source at a rate of 250 μL/h, and 128 single scans were conducted to acquire each mass spectrum.

DNA and RNA sequencing and analysis

Genomic DNA was extracted by phenol, chloroform and isoamyl alcohol method, followed by quality measurement using NanoDrop ND-1000 spectrophotometer (Thermo Fisher Scientific, Waltham, MA, USA). RNA was extracted using TRIzol method (Invitrogen, USA), and cDNA was generated using a SuperScript™ First Strand Synthesis System with random primers, followed by synthesis of the second-strand cDNA using RNase H and DNA polymerase I. DNA and cDNA amplification of the bacterial 16S rRNA genes (V4–V5 region) was performed using the forward primer 515F (5'-GTGCCAGCMGCCGCGGTAA-3') and the reverse primer 907R (5'-CCGTCAATTCMTTTRAGTTT-3') (Xie et al., 2020). Thermal-cycling were follows: initial denaturation at 98°C for 1 min, followed by 30 cycles of denaturation at 98°C for 10 s, annealing at 50°C for 30 s, and elongation at 72°C for 60 s, with a final extension at 72°C for 5 min. Sequencing libraries were generated using an NEB Next® Ultra™ DNA Library Prep Kit (NEB, USA) for Illumina following the manufacturer's recommendations, and index codes were added. Library quality was assessed on a

Qubit@ 2.0 Fluorometer (Thermo Scientific, USA) and Agilent Bioanalyzer 2100 system (USA). Finally, the library was sequenced on an Illumina MiSeq platform (Shanghai Personal Bitechnology Co., Ltd), generating 450 bp paired end reads that were then combined using FLASH software (V1.2.7, http://ccb.jhu.edu/software/FLASH/). Raw data were first quality-filtered with QIIME to remove reads that did not meet the desired quality (sequence quality value ≥ Q20, length ≥ 150 bp) (Caporaso et al., 2010). Chimeras were removed using the Chimera Slayer algorithm in MOTHUR (Edgar et al., 2011). Operational taxonomic units (OTUs) were clustered with a 97% similarity cutoff using UPARSE software (UPARSE, v7.0.1001, http://drive5.com/uparse/). The OTUs were taxonomically classified based on the SILVA database (Version 132).

Structural equation models

We constructed two structural equation models (SEMs) to evaluate the contribution of microbial community structure to the changes in bioavailability of DOM components (Figure S6). In our models, we assumed that the utilization of labile DOM (LDOM, C2) and semi-labile DOM (SLDOM, C3) by the microbes influenced the composition of microbial communities, which in turn affected the quantity, composition and diversity of RDOM (C4). Since the SEMs are only for recursive models, we selected two models for the SEMs; one assumed that the composition of active community drives the quantity, diversity and composition of RDOM (Figure S6A), and the other one assumed that composition of total community is the driver (Figure S6). The composition of

microbial community and SPE-DOM is represented by first axis of principal coordination analysis (Figure S9), which explains the variation of active and total microbial community, and SPE-DOM of 50.62%, 43.36% and 40.35%, respectively. Compared to total microbial communities, the SEMs with active microbial communities have a lower Akaike information criterion (AICc, 39.576 vs 41.416), where the lower AIC indicates a better model that generated by the SEM. This result suggested the active prokaryotes have a higher contribution to DOM transformation compared to total prokaryotes.

The SEMs demonstrated the influence of active degradation of bioavailable DOM by prokaryotes on RDOM pool. LDOM (C2 component) and SLDOM (C3 component) impacted the active microbial community composition. Standardized coefficient (β) was calculated based on the linear regression between variables to describe the relative strengths of predictors to responses. LDOM showed a greater influence than SLDOM on composition of the active microbial communities (β = -0.9799 vs -0.3693). Thereafter, the composition of active microbial communities impacted the composition of DOM (β = 0.7759), and quantity of RDOM with the most significant parameter (β = 0.9713). This result suggested that the composition of the active microbial community contributes more to RDOM accumulation than to altering the composition of SPE-DOM. In comparison, the standardized coefficient of the relation between composition of total community and quantity and composition of RDOM was lower than ones of active community (β = 0.8852 and 0.7324, Figure S6). Overall, the active microbial composition played a more critical role than the total microbial community in the

transformation from LDOM to RDOM. Therefore, we focused on active microbes in the following network analysis.

Interactions among FDOM component, diversity of SPE-DOM and active microbes

The relations among the TOC, FDOM, inorganic nitrogen nutrient, and chemical diversity of SPE-DOM molecules with the OTUs of the active microbial community were evaluated using Spearman's rank correlation (Figure S7). The TOC concentrations (201 links), C2 and C4 FDOM components (194 and 267 links, respectively), and chemodiversity indexes $D_F(C)$ (161 links) revealed a high degree of association with active OTUs. It is notable that the C1, C2 and NH_4 shared 17 active OTUs, which were dominated by four Alphaproteobacterial and four Gammaproteobacterial OTUs, as well as one active OTU belonging to Thaumarchaeota (Figure S7). These results suggested that importance of the NH_4^+ mediated by microorganisms in the transformation of liable C1 and C2 components.

Large complex and separated small subnetworks among the active prokaryotes and MF

The large complex network contained 308 OTUs and 687 MFs (Figure 5). The dominant OTUs within this complex subnetwork belonged to Gammaproteobacteria (40.58%), Alphaproteobacteria (12.66%), Flavobacteriia (26.30%) and SAR202 clade within the Chloroflexi (7.47%). These classes showed different average number of links

to the MFs, 11.66 links for Gammaproteobacteria, 9.23 for Alphaproteobacteria, 10.16 for Flavobacteria, and 12.82 for SAR202 clade, which suggested their different life strategies and metabolic capacities (Figure 5B). The total relative abundance of these active microbes generally decreased over the course of the experiment except for day 20 (Figure S13). The proportion of CHO, CHNO, CHOS and CHNOS were 46.29%, 37.55%, 15.43% and 0.73%, respectively (Table S4). In contrast, the MFs showed a relatively narrow range of m/z values from 221.12 to 440.08 with a small average value of 332.31 ± 44.13 compared to those of MFs in all networks.

The 88 subnetworks were comprised of an average 53 nodes (2 to 443 nodes) while within the selected 44 subnetworks there was an average of 96 nodes (Figure 6). Similar to the large and complex subnetwork, the dominant OTUs belonged to Gammaproteobacteria (36.34%), Alphaproteobacteria (14.17%) and Flavobacteriia (17.59%).

Networks among the total prokaryotes (DNA level) and MF

We also constructed networks based on the total OTUs and MFs to evaluate the relationships among the total prokaryotes and organic molecules in the treatment incubation (Figure S10). Only the correlation with p value of < 0.000678 was kept. Here, we obtained one large and complex subnetwork, and 52 small subnetworks with more than 9 nodes. In these subnetworks, the node indicates DOM molecules or total OTUs, where the edges between them indicate the significantly positive or negative correlation. This network contained 2,071 OTUs and 2,776 MFs. The OTUs were

dominated by the Alpha- and Gammaproteobacteria (31.79% and 14.78%), Acidimicrobiia (12.80%) and Flavobacteriia (12.13%). The CHNO and CHO were the major chemical molecular groups (43.55% and 31.45%). The range of m/z values of these MFs was from 209.1 to 524.2 (421.6 ± 76.1). Generally, the result is similar with the networks between active OTUs and MFs.

Particle organic matter composition

With the physical disruption of the *Synechococcus* cells, additional particle organic matter (POM) was added to the treatment microcosms. POM was dominated by fatty acids, aromatics, phytol, alkenes and alkanes (Figure S11), and mostly metabolized during the phase I. In addition, considering the system parameters, closed system and half year incubation, the mentioned recalcitrant organic molecules here should be the environmental context-dependent recalcitrant DOC (referred as RDOCt) (Jiao et al., 2014).

Composition of total and active communities of particle-associated microbes

The particle-associated microbial communities (size fraction > 3 μm) were dominated by Acidimicrobiia, Flavobacteriia, SAR202 clade (within Chloroflexi), Planctomycetacia, Alphaproteobacteria and Gammaproteobacteria in both control and treatment microcosms (Figure S3), which is consistent with our previous study (Xie et al., 2020).

The nonmetric multiple dimensional scaling (NMDS) analysis revealed a clear

separation of both total and active microbial communities among the different incubation times (Figure S12), which indicated the variation in structure of microbial communities along the incubation time. NMDS analysis also showed that the particle-associated microbial communities were clustered from free-living microbial communities (size fraction > 0.22 μm and < 3 μm) and total microbial communities from no size-fraction sample in control microcosm, suggesting the different trophic strategies between particle-associated and free-living microbial communities. In addition, the closely clustering between free-living and no size fraction microbial communities is in a line with the dominance of the free-living bacteria and the low proportion of the particle-associated bacteria in the environment (generally accounted for less than 20% of total bacteria during phytoplankton blooms, and even less in natural seawaters) (Azam et al., 1983; Ghiglione et al., 2007; Simon et al., 2002). However, the free-living microbial communities were relatively independent from no size fraction communities at RNA level compared to those at DNA level. This indicates that the addition of SOM remarkably affected microbial growth and activities. Therefore, variations of active microbial communities are better to reflect environmental microbial metabolic conditions on a fine scale compared to ones of total microbial communities.

References

Azam, F., Fenchel, T., Field, J.G., Gray, J.S., Meyerreil, L.A. and Thingstad, F. 1983. The ecological role of water-column microbes in the sea. Mar Ecol Prog Ser 10(3), 257-263.

Broek, T.A.B., Bour, A.L.B., Ianiri, H.L., Guilderson, T.P. and McCarthy, M.D. 2019. Amino acid enantiomers in old and young dissolved organic matter: Implications for a microbial nitrogen pump. Geochim Cosmochim Ac 247, 207-219.

Caporaso, G.J., Kuczynski, J., Stombaugh, J., Bittinger, K., Bushman, F.D., Costello, E.K., Fierer, N., Peña, A., Goodrich, J.K., Gordon, J.I., Huttley, G.A., Kelley, S.T., Knights, D., Koenig, J.E., Ley, R.E., Lozupone, C.A., McDonald, D., Muegge, B.D., Pirrung, M., Reeder, J., Sevinsky, J.R., Turnbaugh, P.J., Walters, W.A., Widmann, J., Yatsunenko, T., Zaneveld, J. and Knight, R. 2010. QIIME allows analysis of high-throughput community sequencing data. Nat Methods 7(5), 335-336.

Edgar, R.C., Haas, B.J., Clemente, J.C., Quince, C. and Knight, R. 2011. UCHIME improves sensitivity and speed of chimera detection. Bioinformatics 27(16), 2194-2200.

Fiore, C.L., Longnecker, K., Soule, M.C.K. and Kujawinski, E.B. 2015. Release of ecologically relevant metabolites by the cyanobacterium Synechococcus elongatusCCMP 1631. Environ Microbiol 17(10), 3949-3963.

Ghiglione, J.F., Mevel, G., Pujo-Pay, M., Mousseau, L., Lebaron, P. and Goutx, M. 2007. Diel and seasonal variations in abundance, activity, and community structure of particle-attached and free-living bacteria in NW Mediterranean Sea. Microb Ecol 54(2), 217-231.

Guillemette, F. and del Giorgio, P.A. 2011. Reconstructing the various facets of dissolved organic carbon bioavailability in freshwater ecosystems. Limnol Oceanogr 56(2), 734-748.

Hu, A., Hou, L. and Yu, C.P. 2015. Biogeography of planktonic and benthic Archaeal communities in a subtropical eutrophic estuary of China. Microb Ecol 70(2), 322-335.

Jiao, N., Robinson, C., Azam, F., Thomas, H., Baltar, F., Dang, H., Hardman-Mountford, N.J., Johnson, M., Kirchman, D.L., Koch, B.P., Legendre, L., Li, C., Liu, J., Luo, T., Luo, Y.W., Mitra, A., Romanou, A., Tang, K., Wang, X., Zhang, C. and Zhang, R. 2014. Mechanisms of microbial carbon sequestration in the ocean future research directions (vol 11, pg 5285, 2014). Biogeosciences 11(19), 5565-5565.

Jiao, N.Z., Herndl, G.J., Hansell, D.A., Benner, R., Kattner, G., Wilhelm, S.W., Kirchman, D.L., Weinbauer, M.G., Luo, T.W., Chen, F. and Azam, F. 2010. Microbial production of recalcitrant dissolved organic matter: long-term carbon storage in the global ocean. Nat Rev Microbiol 8(8), 593-599.

Jørgensen, B.B. 1978. A comparison of methods for the quantification of bacterial sulfate reduction in coastal marine sediments. Geomicrobiology Journal 1(1), 29-47.

Kellerman, A.M., Kothawala, D.N., Dittmar, T. and Tranvik, L.J. 2015. Persistence of dissolved organic matter in lakes related to its molecular characteristics. Nat Geosci 8(6), 454-U452.

Koch, B.P. and Dittmar, T. 2006. From mass to structure: an aromaticity index for high-resolution mass data of natural organic matter. Rapid Commun Mass Sp 20(5), 926-932.

Koch, B.P. and Dittmar, T. 2016. From mass to structure: an aromaticity index for high-resolution mass data of natural organic matter (vol 20, pg 926, 2006). Rapid Commun Mass Sp 30(1), 250-250.

Ksionzek, K.B., Lechtenfeld, O.J., McCallister, S.L., Schmitt-Kopplin, P., Geuer, J.K., Geibert, W. and

Koch, B.P. 2016. Dissolved organic sulfur in the ocean: Biogeochemistry of a petagram inventory. Science 354(6311), 456-459.

Kuypers, M.M.M., Marchant, H.K. and Kartal, B. 2018. The microbial nitrogen-cycling network. Nat Rev Microbiol 16(5), 263-276.

Landa, M., Cottrell, M.T., Kirchman, D.L., Kaiser, K., Medeiros, P.M., Tremblay, L., Batailler, N., Caparros, J., Catala, P., Escoubeyrou, K., Oriol, L., Blain, S. and Obernosterer, I. 2014. Phylogenetic and structural response of heterotrophic bacteria to dissolved organic matter of different chemical composition in a continuous culture study. Environ Microbiol 16(6), 1668-1681.

Ma, X., Coleman, M.L. and Waldbauer, J.R. 2018. Distinct molecular signatures in dissolved organic matter produced by viral lysis of marine cyanobacteria. Environ Microbiol 20(8), 3001-3011.

Martin-Cuadrado, A.B., Rodriguez-Valera, F., Moreira, D., Alba, J.C., Ivars-Martinez, E., Henn, M.R., Talla, E. and Lopez-Garcia, P. 2008. Hindsight in the relative abundance, metabolic potential and genome dynamics of uncultivated marine archaea from comparative metagenomic analyses of bathypelagic plankton of different oceanic regions. Isme Journal 2(8), 865-886.

Mentges, A., Feenders, C., Seibt, M., Blasius, B. and Dittmar, T. 2017. Functional Molecular Diversity of Marine Dissolved Organic Matter Is Reduced during Degradation. Frontiers in Marine Science 4.

Moran, M.A. and Durham, B.P. 2019. Sulfur metabolites in the pelagic ocean. Nat Rev Microbiol 17(11), 665-678.

Morando, M. and Capone, D.G. 2018. Direct Utilization of Organic Nitrogen by Phytoplankton and Its Role in Nitrogen Cycling Within the Southern California Bight. Front Microbiol 9.

Orsi, W.D., Smith, J.M., Liu, S.T., Liu, Z.F., Sakamoto, C.M., Wilken, S., Poirier, C., Richards, T.A., Keeling, P.J., Worden, A.Z. and Santoro, A.E. 2016. Diverse, uncultivated bacteria and archaea underlying the cycling of dissolved protein in the ocean. Isme Journal 10(9), 2158-2173.

Simon, M., Grossart, H.P., Schweitzer, B. and Ploug, H. 2002. Microbial ecology of organic aggregates in aquatic ecosystems. Aquat Microb Ecol 28(2), 175-211.

Xie, R., Wang, Y., Chen, Q., Guo, W., Jiao, N. and Zheng, Q. 2020. Coupling Between Carbon and Nitrogen Metabolic Processes Mediated by Coastal Microbes in Synechococcus-Derived Organic Matter Addition Incubations. Front Microbiol 11.

Xie, W., Luo, H., Murugapiran, S.K., Dodsworth, J.A., Chen, S., Sun, Y., Hedlund, B.P., Wang, P., Fang, H., Deng, M. and Zhang, C.L. 2018. Localized high abundance of Marine Group II archaea in the subtropical Pearl River Estuary: implications for their niche adaptation. Environ Microbiol 20(2), 734-754.

Zhao, Z., Gonsior, M., Schmitt-Kopplin, P., Zhan, Y.C., Zhang, R., Jiao, N.Z. and Chen, F. 2019. Microbial transformation of virus-induced dissolved organic matter from picocyanobacteria: coupling of bacterial diversity and DOM chemodiversity. Isme Journal 13(10), 2551-2565.

Zubkov, M.V., Tarran, G.A., Mary, I. and Fuchs, B.M. 2008. Differential microbial uptake of dissolved amino acids and amino sugars in surface waters of the Atlantic Ocean. J Plankton Res 30(2), 211-220.

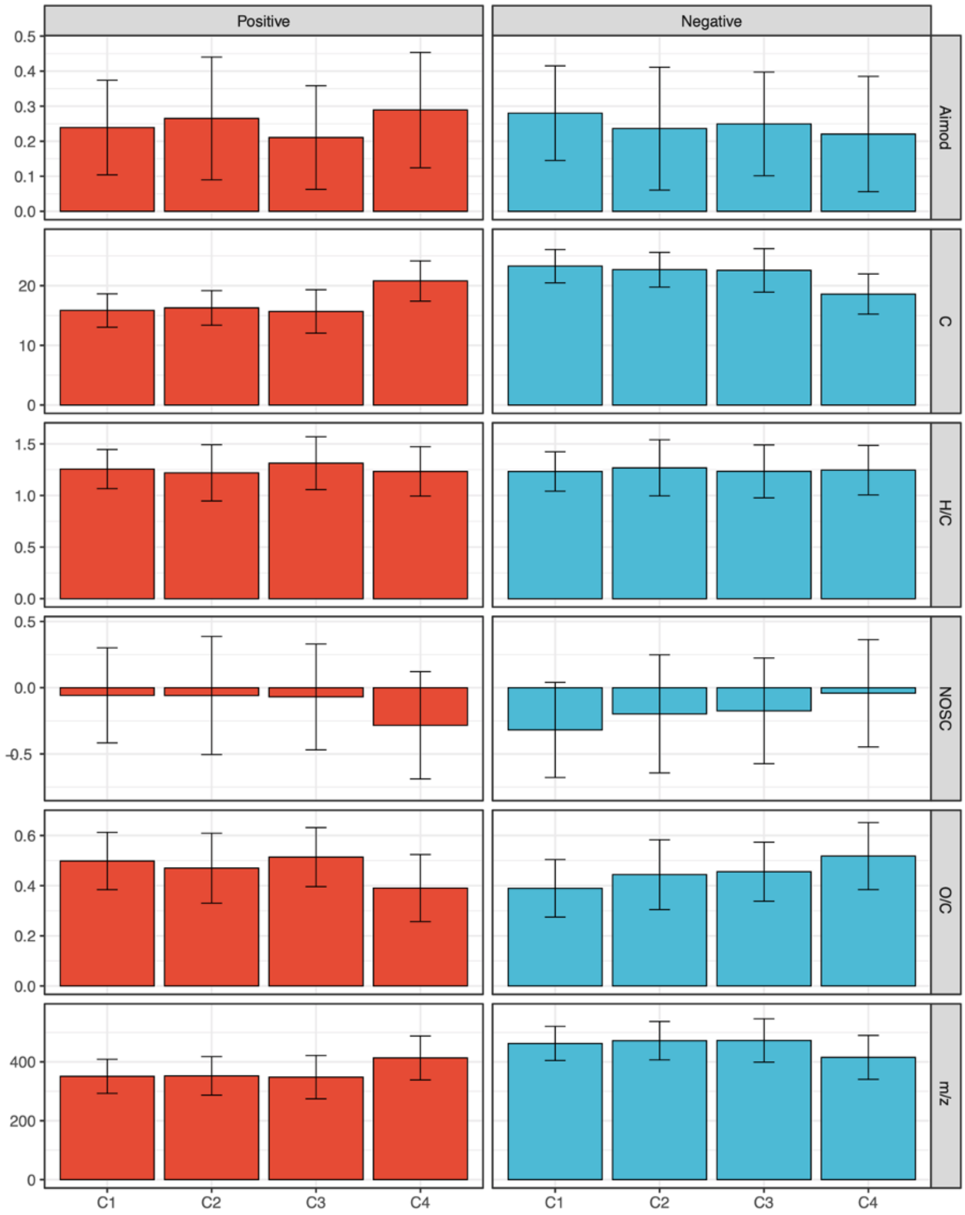

Figure S1 Average properties of molecular formulas of SPE-DOM that correlated with FDOM components. Left and right panels show the molecular formulas that positively and negatively correlated with C1, C2, C3 and C4 FDOM component, respectively. C represents carbon atom number; H/C represents the ratio of hydrogen atom number to carbon atom number; NOSC represents nominal oxidation states of carbon; O/C represents ratio of oxygen atom number to carbon atom number; m/z represents the mass-to-charge ratio.

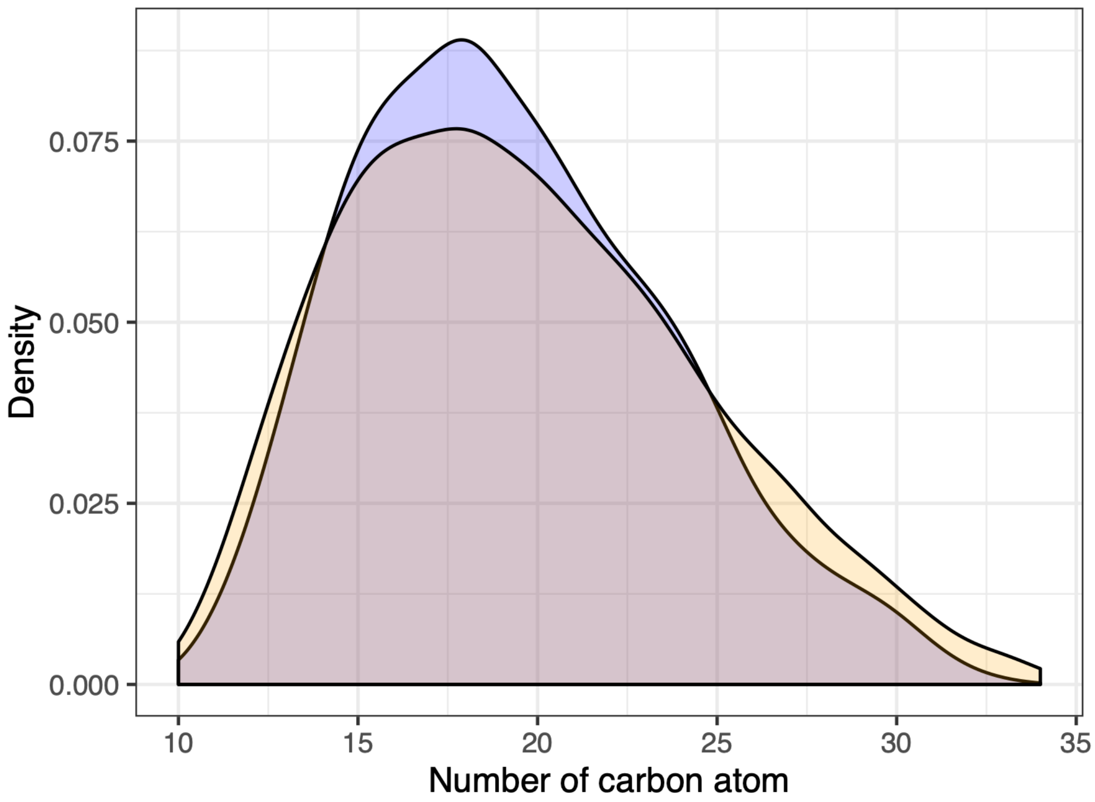

Figure S2 Distribution of the number of carbon atoms in samples from day 0 (purple) and day 180 (yellow) in Synechococcus-derived organic matter addition microcosm. The significance of difference of distribution between two samples was tested by Kolmogorov-Smirnov test, D = 0.040 and p value = 0.012.

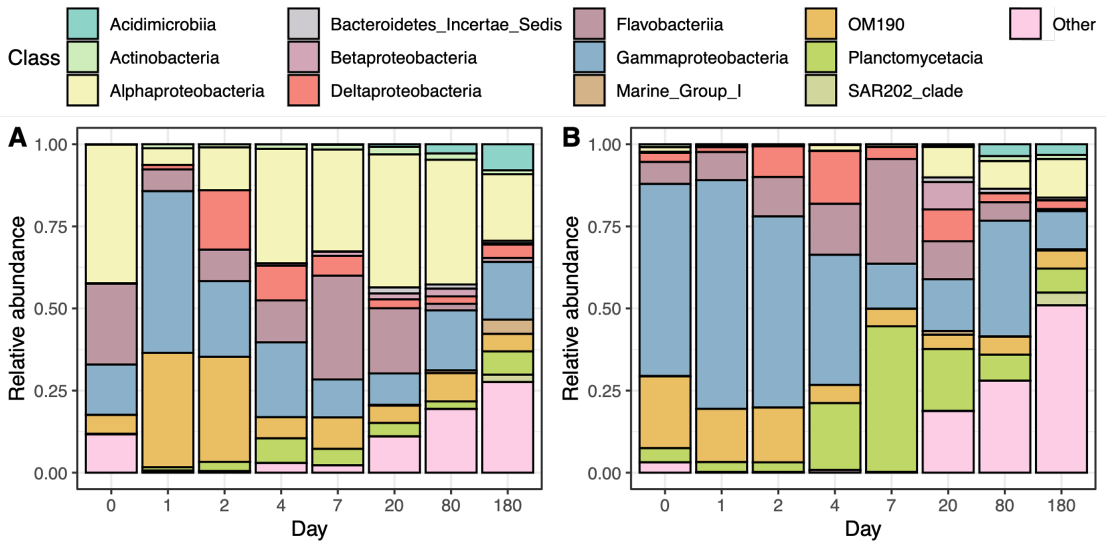

Figure S3 Structure of total (A) and active (B) particle-associated microbial communities (> 3 μm size fraction) at class level in the treatment incubation over the course of the incubation.

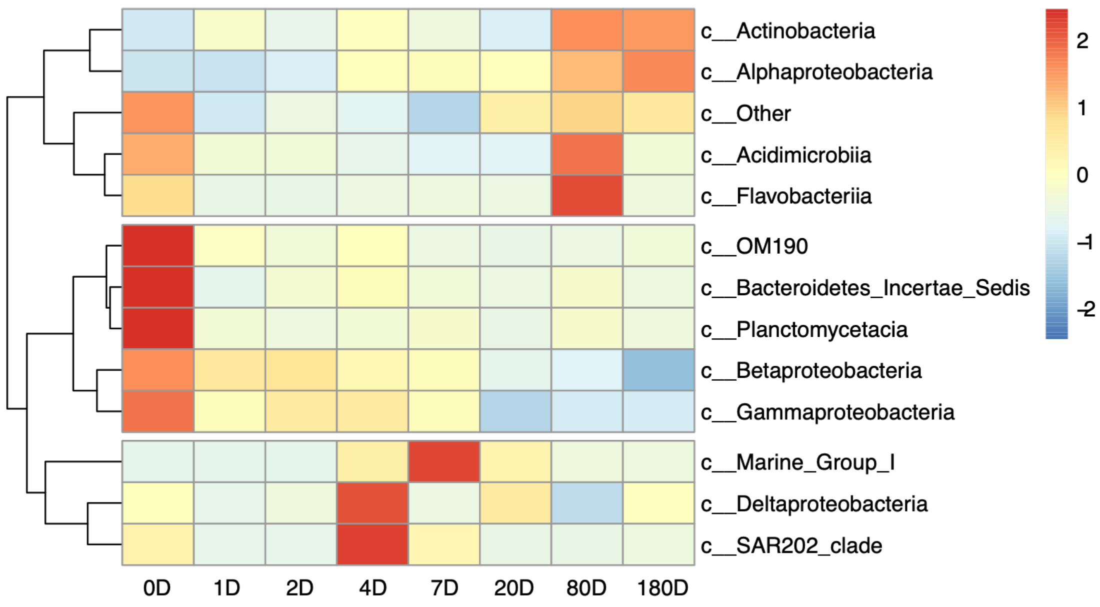

Figure S4 Coarse 16S rRNA:rDNA ratio of bacteria and archaea at class level in treatment microcosms. The color of heatmap shows the normalized ratio.

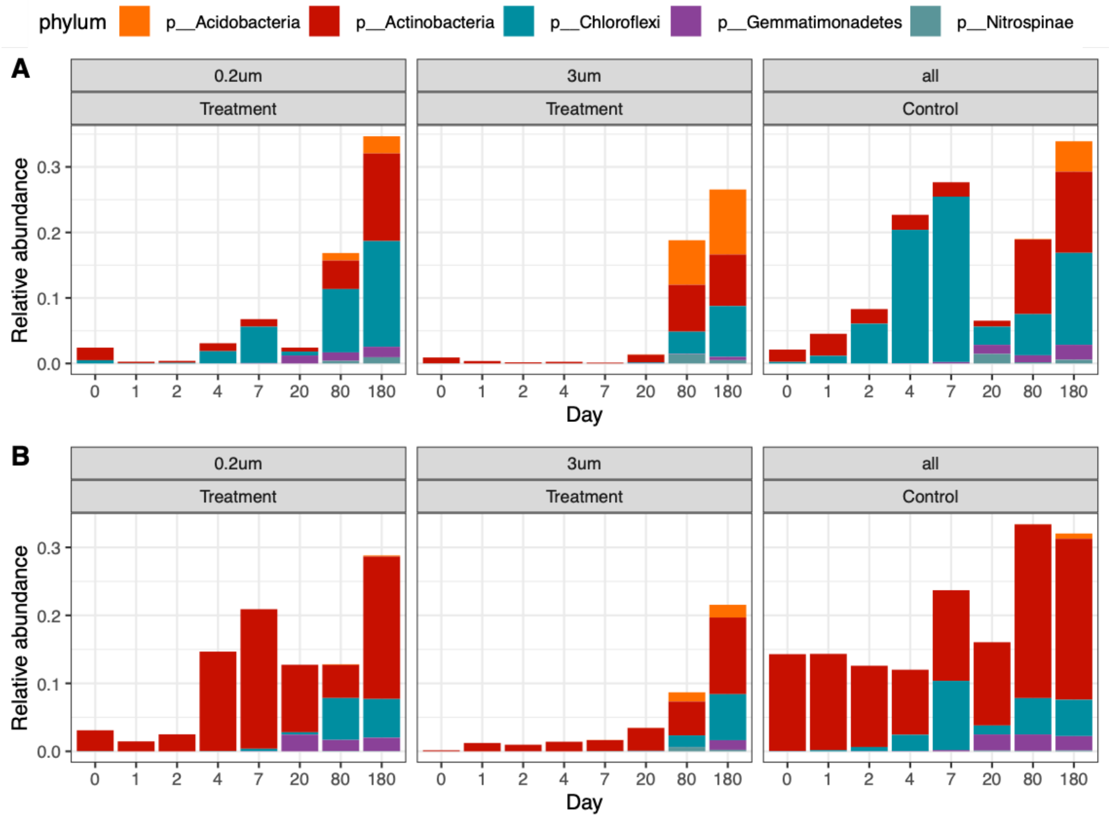

Figure S5 Rare species (phylum level) in early phase at RNA (A) and DNA (B) level that increased along the experiment. T represents the treatment, while C represents the control. 0.2um represents the microbial communities with a size fraction of >0.22 µm and <3 µm, which indicates the free-living microbes in the SOM addition treatment; 3um represents the microbial communities with the size fraction of >3 µm, which indicates the particle-associated microbes in the SOM addition treatment; and the all represents the no size fraction sample in the control.

A

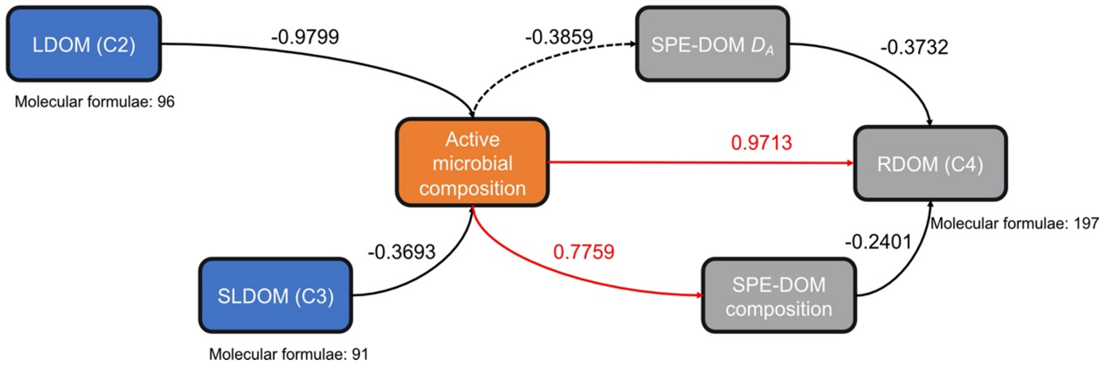

B

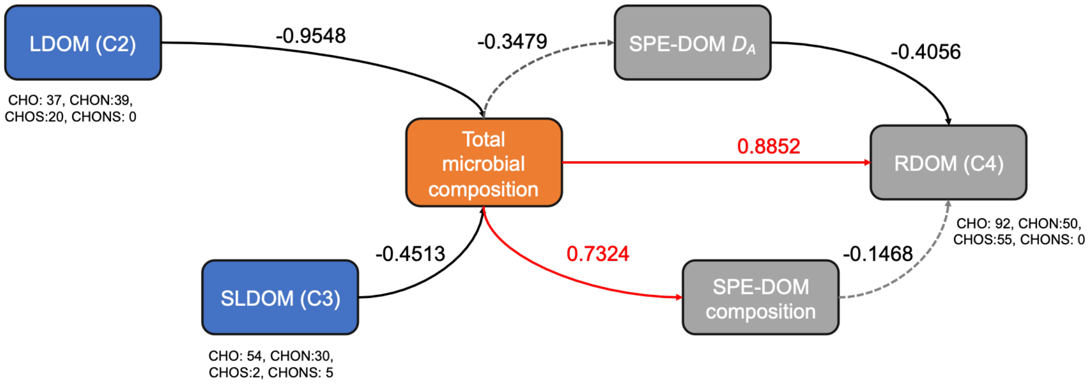

Figure S6 Structural equation model of active (A) and total (B) microbial community composition, SPE-DOM composition, DA of SPE-DOM and FDOM C2, C3 and C4 component. Solid line indicates significant linear regression with P-value < 0.05, while dashed line indicates P-value ≥ 0.05. The number of MFs that significantly correlated with C2, C3 and C4 component were shown based on Spearman correlation with p value < 0.01. (A) Fisher's C = 11.146 with P-value = 0.674; AICc = 41.146 and no missing variable. (B) Fisher's C = 9.576 with P-value = 0.792; AICc = 39.576 and no missing variable.

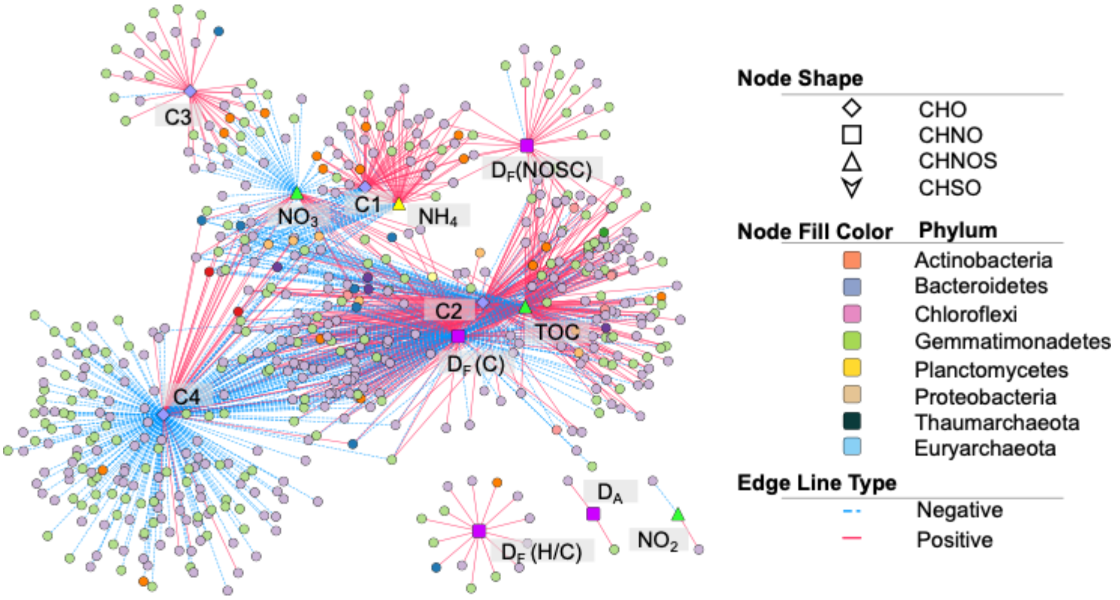

Figure S7 Association among OTUs and FDOM components, total organic carbon and nitrogen nutrients and diversity indexes of SPE-DOM. Only the correlation with p value < 0.01 is kept. Square indicate FDOM component, total organic carbon, nitrogen nutrient or DOM diversity index. C1, C2, C3 and C4 indicates the relevant FDOM component. $D_F(C)$, $D_F(H/C)$, $D_F(NOSC)$ and D_A indicates the DOM functional diversity based on the carbon atom number, H/C ratio and NOSC, and abundance-based diversity, respectively. Circle indicates a microbial OTU with color representing the phylum. Blue and dashed line indicates the negative correlation, while red and solid line indicates positive correlation.

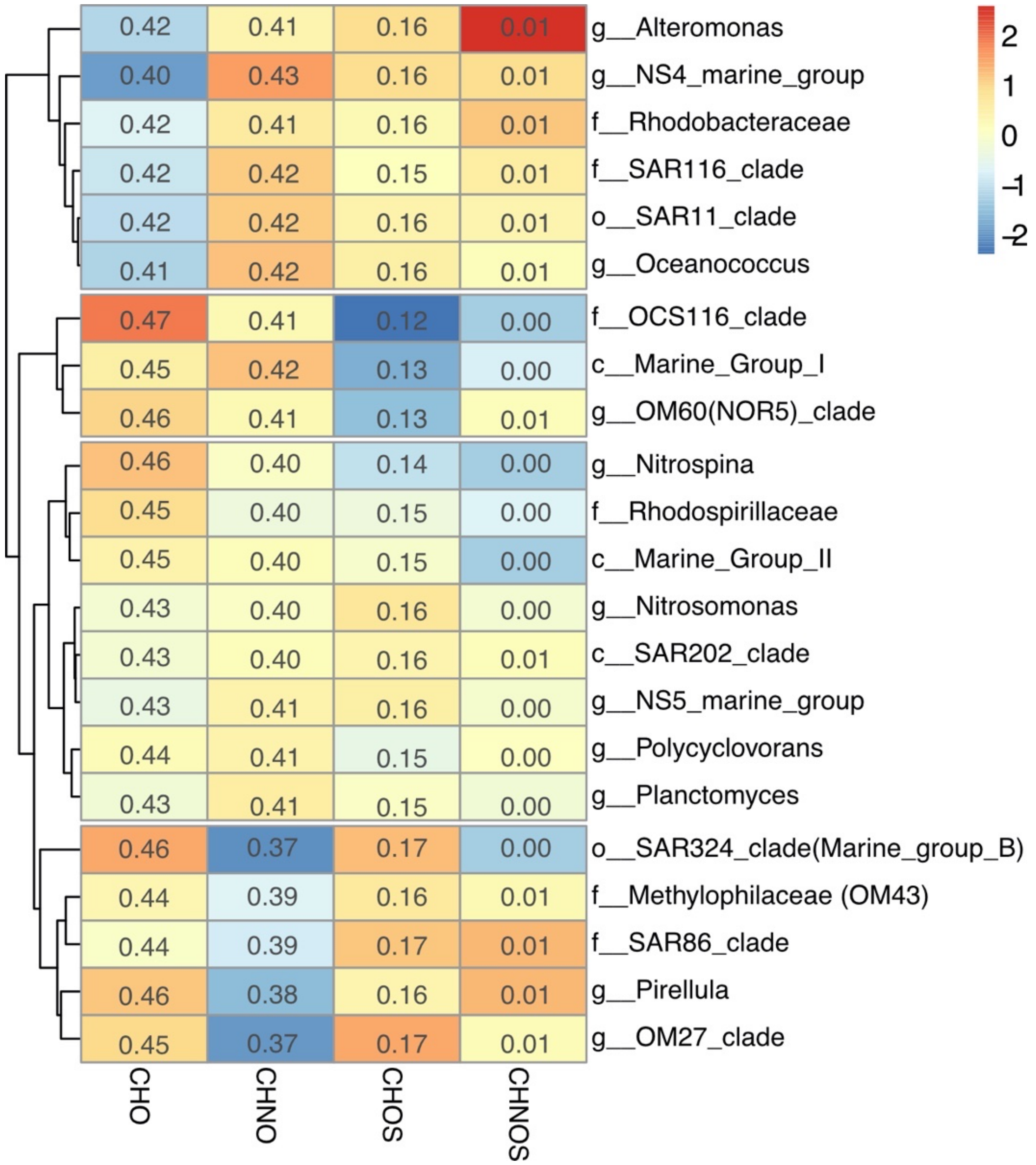

Figure S8 Clustering of proportion of CHO, CHNO, CHOS, and CHNOS associated with microbes. Color bar shows the normalized proportion by the column, while the number shows the proportion of each molecular formula group. F indicates the family, o indicates the order, and g indicates the genus.

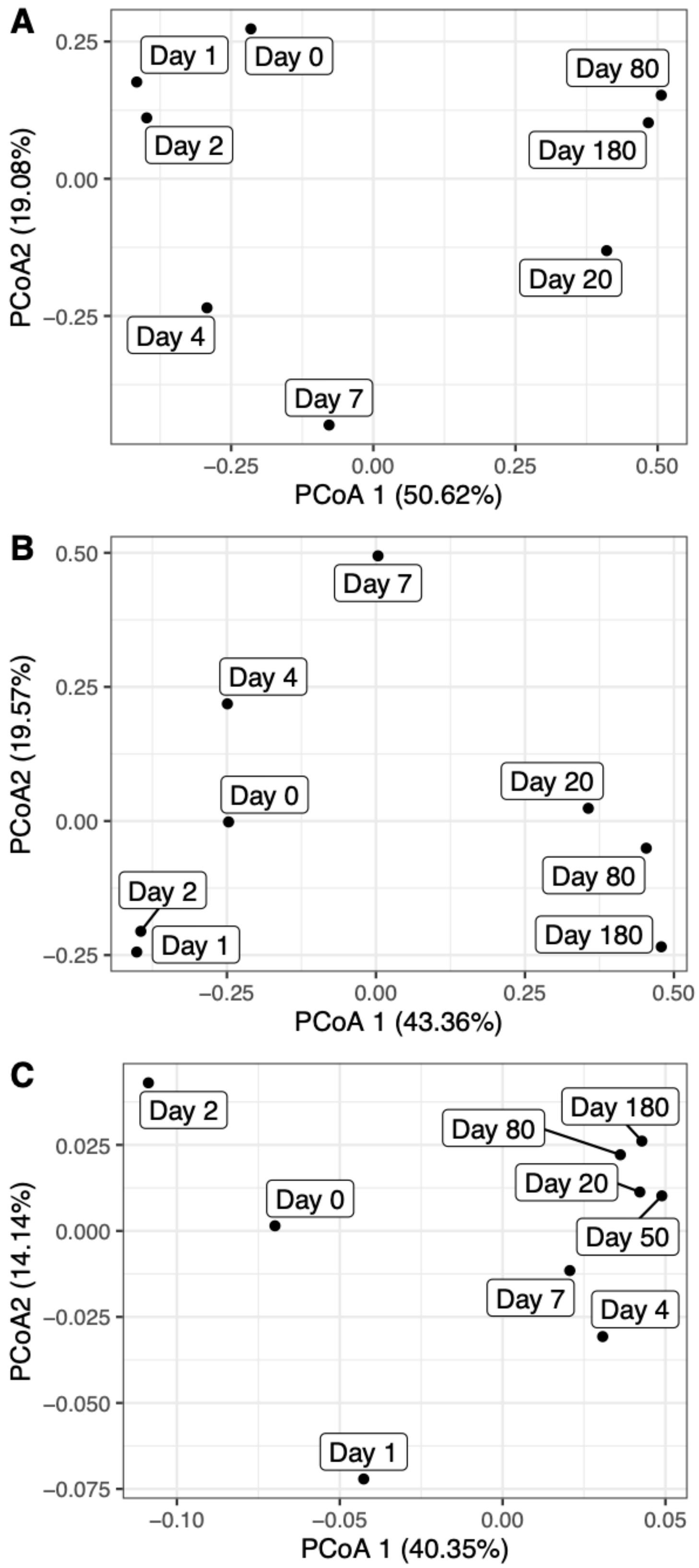

Figure S9 Principal coordination analysis of total, active microbial community (A and B) and SPE-DOM (C) in treatment.

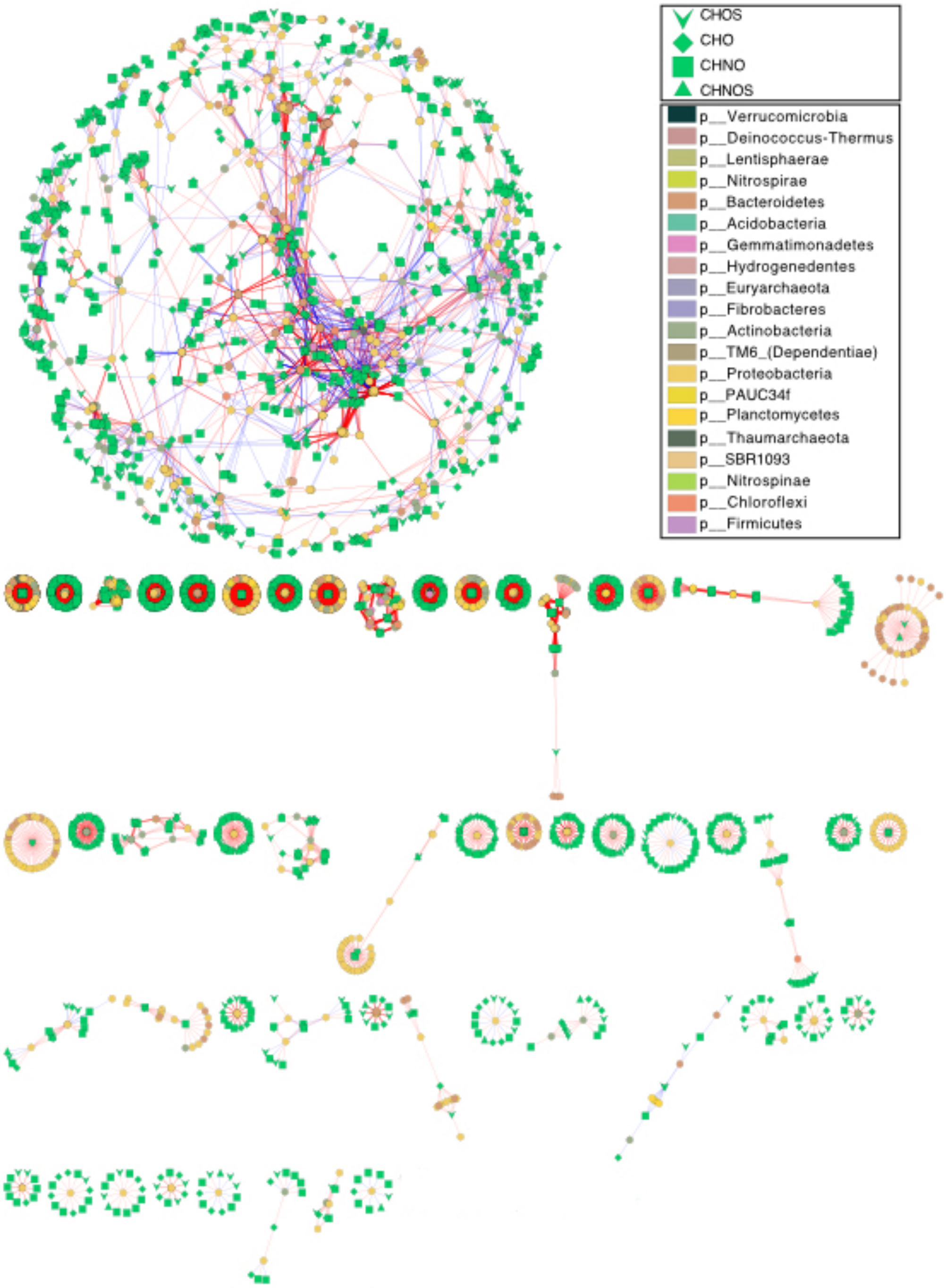

Figure S10 Networks based on the spearman correlation between the total prokaryotes and molecular formulas in the treatment. Circle represents the active OTU with color indicating phylum, while diamond, square, triangle and arrow represent CHO, CHNO, CHNOS, and CHOS, respectively. The blue and red edge indicate the negative and positive correlation with p value < 0.000678 separately.

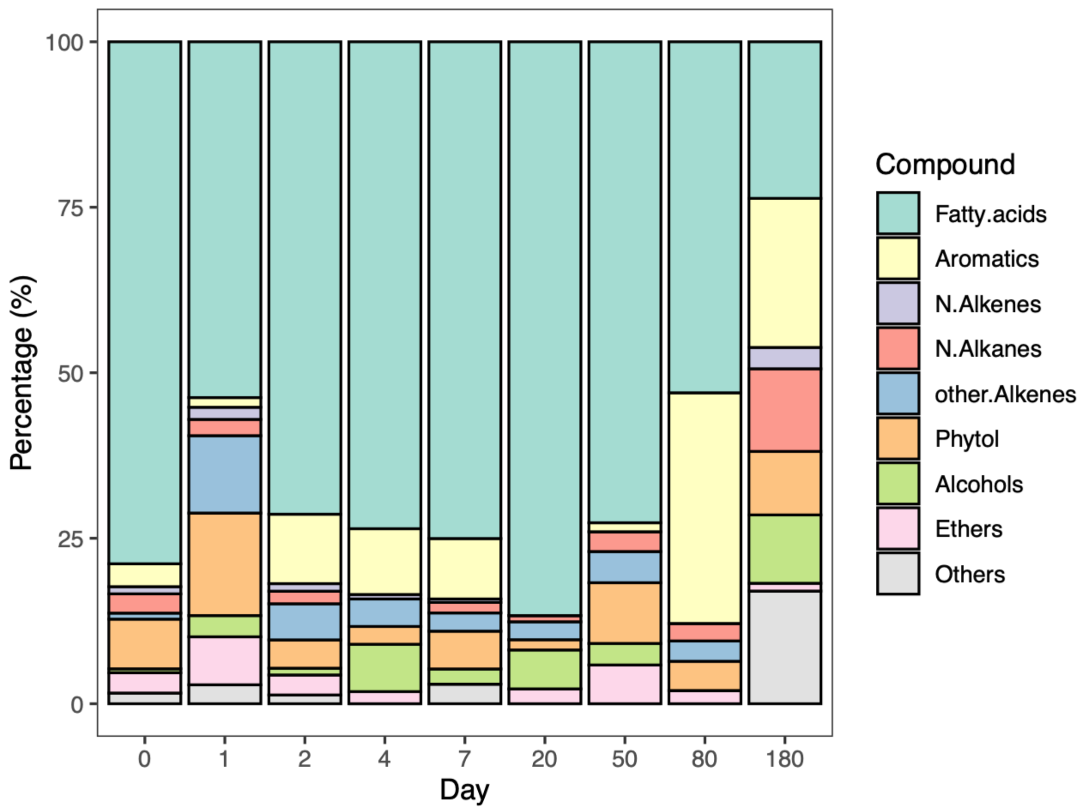

Figure S11 Particle organic matter composition in treatment microcosms.

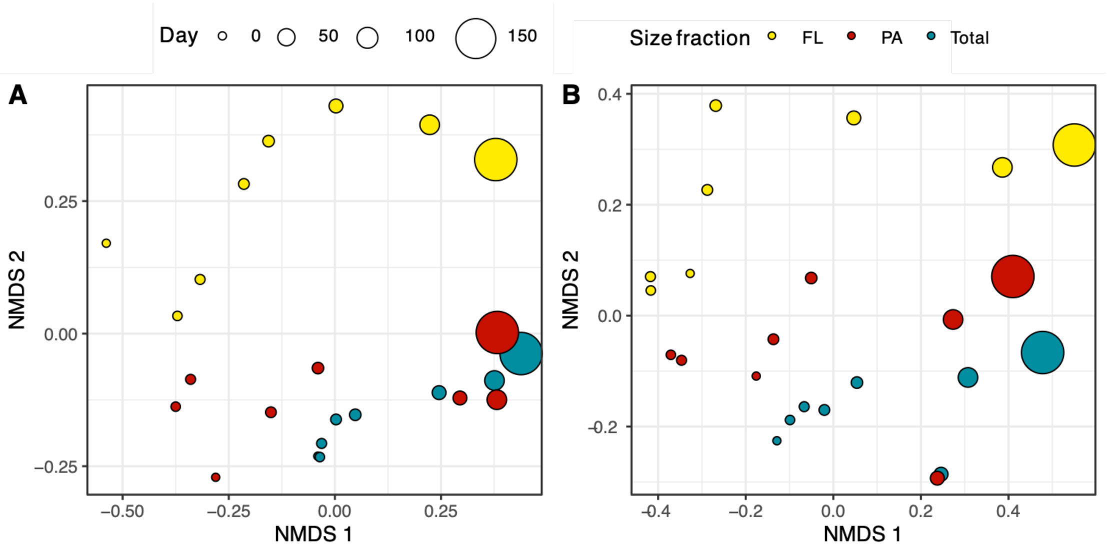

Figure S12 Nonmetric multidimensional (NMDS) analysis of total (A) and active (B) microbial communities. FL indicates the sample of size fraction > 0.22 μm and < 3 μm in treatment, while PA indicates the sample of size fraction > 3 μm in treatment. Total indicates the sample of size > 0.22 μm in control. Dot size indicates the incubation day.

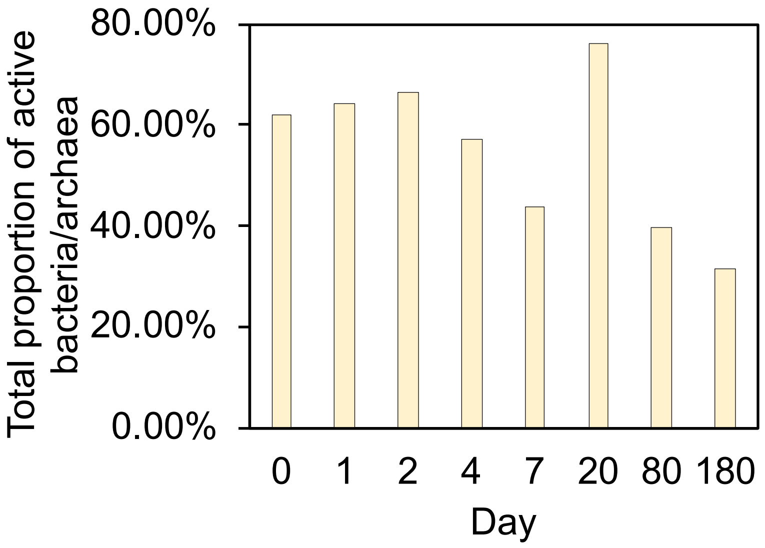

Figure S13 Total proportion of active bacteria/archaea in the large complex network along the experiment.

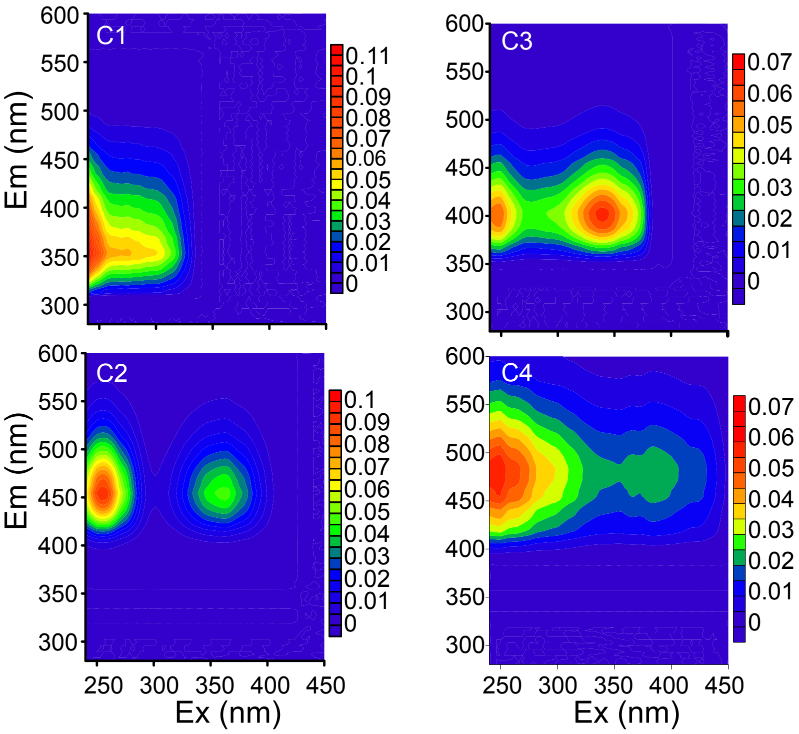

Figure S14 Component of fluorescence dissolved organic carbon defined by the PARAFAC model.

Table S1 Chemical properties of SPE-DOM in Synechococcus-derived organic matter addition microcosms and control microcosms

Day	MF no.[a]	CHO	CHNO	CHOS	CHNOS	m/z(WM)[b]	AI_{mod} (WM)
Total[c]							
	5652	38.02%	42.62%	16.51%	2.85%	NA	NA
Treatment							
0	2932	47.95%	37.72%	13.68%	0.65%	408.19	0.22
1	2469	49.53%	36.01%	14.46%	0.00%	403.07	0.21
2	2551	49.16%	35.44%	15.09%	0.31%	415.36	0.22
4	3434	43.80%	39.46%	15.46%	1.28%	402.52	0.21
7	3648	43.53%	40.49%	14.67%	1.32%	403.90	0.22
20	3799	41.64%	41.85%	14.61%	1.90%	408.44	0.21
50	3672	44.12%	39.92%	15.11%	0.84%	406.25	0.21
80	3549	44.15%	40.38%	14.26%	1.21%	407.77	0.22
180	3609	43.70%	40.76%	14.16%	1.39%	407.14	0.22
Control							
0	3322	45.42%	37.66%	15.65%	1.26%	420.49	0.22
180	3440	46.40%	39.56%	13.46%	0.58%	418.51	0.23

[a]MF no. indicates the number of the detected molecular formulas;

[b]m/z(WM) and AI_{mod} (WM) indicate the sum of weighted m/z and AI_{mod} by MF intensity in each sample, respectively;

[c]Total indicates the MF detected from all the samples.

Table S2 Number of MF-central and OTU-central subnetworks

Day	Phase	MF-central (log_fold >= 1)	OTU-central (log_fold <= -1)
0,1,4,7	Early (phase I)	14	16
20,80,180	Late (phase II or III)	1	15

Phase was determined based on day the relative abundance of active OTUs reached their peak during the experiment.

Table S3 Proportion and molecular formulae number of CHO, CHNO, CHOS and CHNOS groups in subnetwork

	CHO	CHNO	CHOS	CHNOS
OTU-central				
O1	45.25% (100)	38.91% (86)	15.84% (35)	0.00% (0)
O2	44.68% (21)	34.04% (16)	19.15% (9)	2.13% (1)
O3	49.06% (26)	39.62% (21)	11.32% (6)	0.00% (0)
O4	30.30% (10)	36.36% (12)	33.33% (11)	0.00% (0)
O5	40.63% (13)	43.75% (14)	15.63% (5)	0.00% (0)
O6	32.43% (12)	48.65% (18)	18.92% (7)	0.00% (0)
O7	30.30% (10)	48.48% (16)	18.18% (6)	3.03% (1)
O8	44.44% (12)	37.04% (10)	18.52% (5)	0.00% (0)
O9	45.45% (10)	40.91% (9)	13.64% (3)	0.00% (0)
O10	45.45% (10)	40.91% (9)	13.64% (3)	0.00% (0)
O11	46.15% (6)	38.46% (5)	15.38% (2)	0.00% (0)
O12	50.00% (7)	14.29% (2)	35.71% (5)	0.00% (0)
O13	54.55% (6)	36.36% (4)	9.09% (1)	0.00% (0)
O14	41.67% (5)	41.67% (5)	16.67% (2)	0.00% (0)
O15	63.64% (7)	36.36% (4)	0.00% (0)	0.00% (0)
O16	60.00% (6)	40.00% (4)	0.00% (0)	0.00% (0)
O17	25.00% (2)	50.00% (4)	25.00% (2)	0.00% (0)
MF-central				

M1	31.25% (10)	56.25% (18)	12.50% (4)	0.00% (0)
M2	50.00% (1)	50.00% (1)	0.00% (0)	0.00% (0)
M3	38.24% (13)	52.94% (18)	8.82% (3)	0.00% (0)
M4	66.67% (2)	33.33% (1)	0.00% (0)	0.00% (0)
M5	37.25% (19)	43.14% (22)	15.69% (8)	3.92% (2)
M6	30.00% (9)	56.67% (17)	10.00% (3)	3.33% (1)
M7	40.00% (4)	40.00% (4)	10.00% (1)	10.00% (1)
M8	44.44% (4)	22.22% (2)	33.33% (3)	0.00% (0)
M9	33.33% (2)	0.00% (0)	66.67% (4)	0.00% (0)
M10	50.00% (2)	25.00% (1)	25.00% (1)	0.00% (0)
M11	25.00% (1)	50.00% (2)	25.00% (1)	0.00% (0)
M12	75.00% (6)	0.00% (0)	25.00% (2)	0.00% (0)
M13	0.00% (0)	50.00% (1)	50.00% (1)	0.00% (0)
M14	40.00% (2)	40.00% (2)	20.00% (1)	0.00% (0)
Small complex				
C1	48.17% (79)	40.85% (67)	10.98% (18)	0.00% (0)
C2	46.67% (63)	35.56% (48)	17.78% (24)	0.00% (0)
C3	45.12% (74)	37.20% (61)	16.46% (27)	1.22% (2)
C4	35.97% (50)	48.92% (68)	14.39% (20)	0.72% (1)
C5	43.75% (35)	38.75% (31)	17.50% (14)	0.00% (0)
C6	38.46% (20)	50.00% (26)	11.54% (6)	0.00% (0)
C7	48.72% (19)	46.15% (18)	5.13% (2)	0.00% (0)

C8	40.00% (10)	44.00% (11)	12.00% (3)	4.00% (1)
C9	42.11% (8)	36.84% (7)	21.05% (4)	0.00% (0)
C10	40.00% (6)	33.33% (5)	26.67% (4)	0.00% (0)
C11	55.56% (10)	27.78% (5)	11.11% (2)	5.56% (1)
C12	53.85% (7)	30.77% (4)	15.38% (2)	0.00% (0)
C13	40.00% (2)	40.00% (2)	0.00% (0)	20.00% (1)

Table S4 Proportion and number of the DOM molecular formulas in the large complex network

	Proportion (Number)
CHO	46.29% (318)
CHNO	37.55% (258)
CHOS	15.43% (106)
CHNOS	0.73% (5)
